# Graph Fourier transform for spatial omics representation and analyses of complex organs

**DOI:** 10.21203/rs.3.rs-3952048/v1

**Published:** 2024-02-15

**Authors:** Yuzhou Chang, Jixin Liu, Yi Jiang, Anjun Ma, Yao Yu Yeo, Qi Guo, Megan McNutt, Jordan Krull, Scott J. Rodig, Dan H. Barouch, Garry Nolan, Dong Xu, Sizun Jiang, Zihai Li, Bingqiang Liu, Qin Ma

**Affiliations:** 1 Department of Biomedical Informatics, College of Medicine, Ohio State University, Columbus, OH 43210, USA.; 2 Pelotonia Institute for Immuno-Oncology, The James Comprehensive Cancer Center, The Ohio State University, Columbus, OH 43210, USA.; 3 School of Mathematics, Shandong University, Jinan 250100, China.; 4 Center for Virology and Vaccine Research, Beth Israel Deaconess Medical Center, Boston, MA 02115, USA.; 5 Department of Pathology, Dana Farber Cancer Institute, Boston, MA 02115 USA.; 6 Department of Pathology, Brigham & Women’s Hospital, Boston, MA 02115, USA.; 7 William Bosworth Castle Professor of Medicine, Harvard Medical School; 8 Ragon Institute of MGH, MIT, and Harvard; 9 Department of Microbiology and Immunology, Stanford University School of Medicine, Stanford, CA 94305, USA.; 10 Department of Pathology, Stanford University School of Medicine, Stanford, CA 94305, USA.; 11 Department of Electrical Engineering and Computer Science, and Christopher S. Bond Life Sciences Center, University of Missouri, Columbia, MO 65211, USA.; 12 Broad Institute of Harvard and MIT, Cambridge, MA 02142, USA.; 13 Department of Microbiology, Harvard Medical School, Boston, MA 02115, USA.

## Abstract

Spatial omics technologies are capable of deciphering detailed components of complex organs or tissue in cellular and subcellular resolution. A robust, interpretable, and unbiased representation method for spatial omics is necessary to illuminate novel investigations into biological functions, whereas a mathematical theory deficiency still exists. We present SpaGFT (Spatial Graph Fourier Transform), which provides a unique analytical feature representation of spatial omics data and elucidates molecular signatures linked to critical biological processes within tissues and cells. It outperformed existing tools in spatially variable gene prediction and gene expression imputation across human/mouse Visium data. Integrating SpaGFT representation into existing machine learning frameworks can enhance up to 40% accuracy of spatial domain identification, cell type annotation, cell-to-spot alignment, and subcellular hallmark inference. SpaGFT identified immunological regions for B cell maturation in human lymph node Visium data, characterized secondary follicle variations from in-house human tonsil CODEX data, and detected extremely rare subcellular organelles such as Cajal body and Set1/COMPASS. This new method lays the groundwork for a new theoretical model in explainable AI, advancing our understanding of tissue organization and function.

Advancements in spatial omics offer a comprehensive view of the molecular landscape within the native tissue microenvironment, including genome, transcriptome, microbiome, T cell receptor (TCR)^1^, epigenome, proteome, transcriptome-protein markers co-profiling, and epigenome–transcriptome co-profiling^2^ (Fig. 1a and Supplementary Fig. 1). These approaches enable the investigation and elucidation of functional tissue units (FTUs)^3^, which are defined as over-represented multicellular functional regions with a unique physiologic function, with both cell-centric and gene-centric approaches. Specifically, cell-centric approaches involve the identification of spatial domains with coherent gene expression and histology^4^, studying cell composition and neighborhoods within specific domains^5–7^, and understanding inter-cellular mechanisms. In parallel, gene-centric approaches characterized FTUs by imputing gene expression^8^ and identifying spatially variable genes (SVG)^9–11^ in a highly complementary manner to cell-centric approaches.

Compared with statistical methods, graph-based methods can efficiently encode and leverage spatial relationships within tissue in spatial omics data representation^12^. We postulate that an FTU can be intuitively considered a graph, its nodes represent spots or cells, and edges connect spatially adjacent or functionally related nodes. Within this representation of FTUs, a binary graph signal (e.g., 0,1), representing discrete two-state information at each node, and cellular or subcellular composition or omics features (e.g., genes) constitute continuous graph signals, encoding a range of values across the graph’s nodes. These graph signals define the FTU’s characterization, connect cell-centric and gene-centric analyses, and offer mutual interpretatibility^13^, through the generation of a graph embedding that harmonizes the graph structure and signal magnitude. Furthermore, while graph-based machine learning methods are available to learn graph embeddings and carry out downstream tasks (e.g., graph classification), they usually rely on an inductive bias (i.e., a hypothesis for a particular question) to train the model^14^. As a result, the produced graph embeddings are either biased or only have applicative power in limited downstream tasks. Therefore, there is an urgent need for a general graph signal representation framework to reveal intricate relations between molecular signatures and FTUs across multiple-resolution scales of spatial omics data.

To achieve this, we present SpaGFT (Spatial Graph Fourier Transform), an unbiased analytical feature representation approach to encode graph signals for representing biological processes within tissues and cells, enabling various downstream analyses and ensuring insightful biological findings. Computationally, it outperformed other tools in identifying SVGs with hundred-fold efficiency and gene expression imputation across human/mouse Visium data. Biologically, SpaGFT identified key immunological areas for B cell maturation processes from human lymph nodes Visium data and further illustrated secondary follicle cellular, morphological, and molecular diversity from exclusively in-house human tonsils CODEX data. Moreover, it has enabled the discovery of extremely rare subcellular structures like the Cajal body and Set1/COMPASS complex based on iterative indirect immunofluorescence image (4i) data^15^, enhancing our understanding of cellular function using spatial omics technologies.

## Results

### SpaGFT unbiasedly represents spatial omics data.

**Figure 1.**
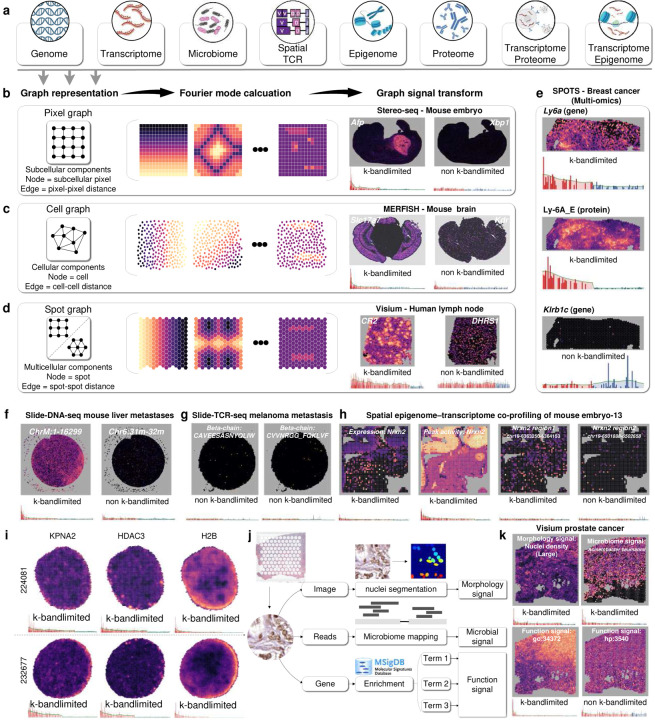
**a.** The panel showcases spatial omics technologies, including single and multi-modality methods. **b-d**, the panels display the calculation of Fourier modes (FM), and the transformation of original graph signals into Fourier coefficients (FC) with different resolutions of technologies. **b.** The figure presents pixel graphs with nodes at the subcellular level and edges denoting short Euclidean distances between connected pixels. This graph represents technologies like stere-seq and most spatial proteomics data, e.g., 4i. The two figures following panel **b** illustrate a k-bandlimited signal (e.g., Afp) and a non-k-bandlimited signal (e.g., Xbp1). **c** and **d**. cell graphs and spot graphs are composed of nodes at the cellular level resolution and multicellular level resolution, respectively, with edges representing short Euclidean distances between nodes in two panels. **e**. the figure exhibits multi-modal data from a technology called SPOTS, which can measure both proteins and genes simultaneously. The k-bandlimited signal shown is for the Ly6a gene and its corresponding protein, while the non-k-bandlimited signal is for the Klrb1c gene. **f**, **g**, **and h**. The panels show examples of signals from Slide-DNA-seq, Slide-TCR-seq, and spatial epigenome–transcriptome co-profiling of mouse embryo-13. **i**. The panel shows subcellular spatial proteome (i.e., 4i) are k-bandlimited signals. **j**. This panel demonstrates data augmentation for sequencing-based spatial transcriptomics (e.g., Visium). The first step of augmentation involves using H&E images and Cellpose for cell segmentation and counting the number of nuclei in each spot. The next step involves mapping reads to the microbiome genome, which then allows for the determination of microbiome abundance. Finally, gene lists (e.g., MSigDB) can be used to calculate the pathway activity score for each spot. **k**. This panel displays the signals mentioned in panel **j**, including cell density, microbiome abundance, and pathway activity.

We summarize current spatially resolved omics as three types of spatial graphs related to the granularity of nodes, ranging from subcellular level (i.e., pixel-level) to broader cellular (i.e., cell-level) and multicellular scales (i.e., spot-level) based on the spatial resolutions (Figs. 1b–k). This granularity can range from subcellular levels to broader cellular and multicellular scales. For example, based on the spatial graph of a spatially resolved transcriptomics (SRT) dataset, the transcriptomic profile of a specific gene is a graph signal and can be represented by the linear combination of its Fourier modes (FMs, Terminology Box 1). To elaborate, a low-frequency FM contributes to a low and smooth graph signal variation, representing a spatially organized pattern, while a high-frequency FM contributes to rapid graph signal variation and usually refers to noises in spatial omics data^16^. For example, if a gene exhibits a spatially organized pattern in SRT data, the Fourier coefficients (FCs) of corresponding low-frequency FMs are more dominant than FCs of high-frequency FMs in the graph Fourier representation. Notably, FMs are associated with graph structure and do not assume any predefined patterns^16^, ensuring flexibility in representing both well-defined and irregular spatial signal patterns. Thus, regardless of single- (Figs. 1b–d, 1f, 1g, 1i), multi-modalities (Figs. 1e and 1h), or augmented features (Figs. 1j and 1k), the spatial omics can be analytically transformed into FCs to quantify the contribution of FMs in the frequency domain^17^, a feature space for enhancing the interpretability and generalizability in downstream analyses.

### SpaGFT identifies spatially variable genes and enhances gene and protein signals.

**Figure 2.**
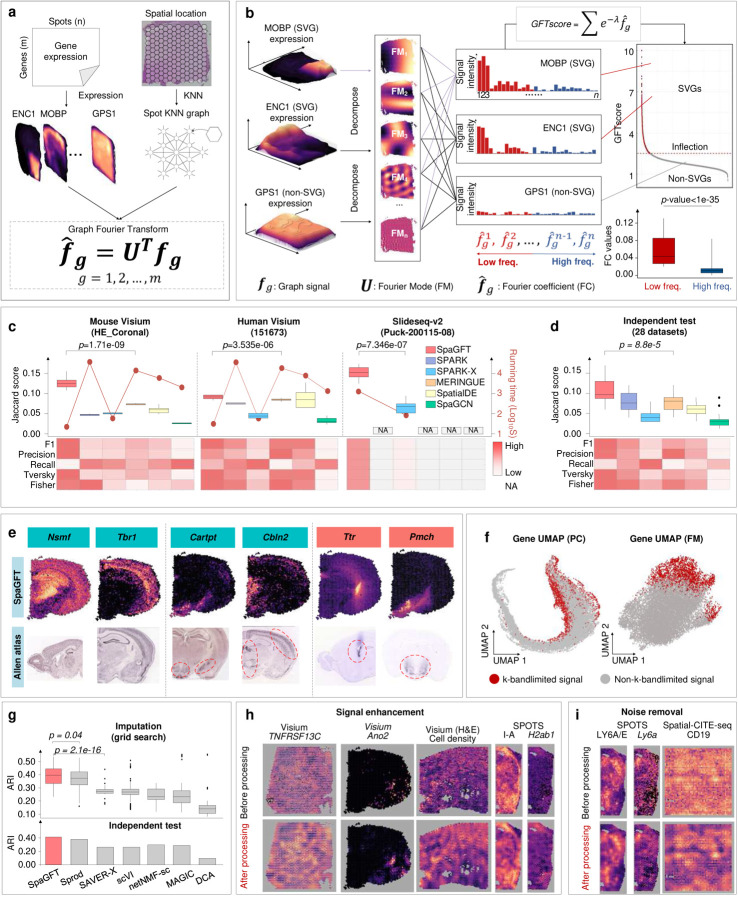
**a.** SpaGFT considers a gene-spot expression count matrix (*m* × *n*) and spatial locations as input data, with *ENC1*, *MOBP*, and *GPS1* listed as examples. A KNN spot graph is generated by calculating the Euclidean distance among spots based on spatial locations between any two spots. Combining gene expressions and spot KNN graph, the graph signal $f_{g}$ of gene g can be projected to a series of FM U and transformed into a frequency signal ${\overset{ˆ}{f}}_{g}$ using a graph Fourier transform. **b**. Two known SVGs (*MOBP* and *ENC1*) and one non-SVG (*GPS1*) are shown as examples. A gene can be decomposed into multiple FMs (a series of periodic signals with gradually faded patterns) and corresponding frequency signals. The FMs can be separated into low-frequency (red) and high-frequency (blue) domains. For each gene, a *GFTscore* was designed to measure the FCs in the low-frequency domain quantitatively. Boxplot in the right-bottom corner shows that the low-frequency FCs of 1,902 SVGs from sample 151673 are significantly higher than high-frequency FCs (with a p-value<1e^−35^ in the Wilcoxon rank-sum test). **c**. The SVG prediction evaluation was compared to five benchmarking tools in terms of reference-based evaluation methods, the Jaccard Index, Tversky Index, Fisher Statistic, Precision, Recall, and F1 score. The running time (seconds in log transformation) of each tool is represented as red lines. In addition, the F1 score, precision, recall, Tversky index, and odds ratio associated with Fisher’s exact test on all parameter combinations for each tool are shown as heatmaps. The Wilcox rank-sum test was used to calculate the p-value for the highest two tools (i.e., parameter combination number N = 16 and N = 53 for SpaGFT and MERINGUE in HE_coronal data; N = 16 and N = 54 for SpaGFT and MERINGUE in 151673 data; N = 16 and N = 18 for SpaGFT and SPARK-X in Puck-200115–08 data). Particularly, regarding larger datasets such as Slide-seqV2, the statistical methods might not be able to identify SVG in a reasonable time, showing NA in this panel. **d**. The parameter combination showing the highest median Jaccard scores among all three benchmark datasets was selected as the default parameter for each tool. Using such a parameter selection, the SVG prediction performance of SpaGFT was compared to those of the five benchmark tools. The black line in each box indicates the median Jaccard score. The statistical test method was the same as panel f to calculate the p-values for the highest two tools (N = 28). **e**. SVG examples that all tools can identify (validated by the ground truth, left panel), uniquely identified by SpaGFT (validated by the ground truth, middle panel), and uniquely identified by SpaGFT (not validated, right panel). If the coronal plane of ISH validation data was unavailable, the sagittal plane was used. **f**. Comparison of the UMAPs obtained using the top 207 PCs (left) and the top 207 FCs (right) of the Mouse Visium data (HE-coronal, 2,702 spots). PCA dimensions were generated directly from the gene-spot expression matrix using Scanpy. Red dots indicate the 2,157 SVGs identified by SpaGFT using default settings, whereas the grey color suggests non-SVGs. Red circles in the ISH data indicate the expression region of the mouse brain. **g**. Boxplot showcases the performance of SVG signal enhancement for grid search (top) and independent test using 151509 (bottom). The y-axis is the ARI value, and the y-axis is the imputation tool name. The statistical test method was the same as panel f to calculate the p-values for the highest two tools (N = 27). **h** and i. The spatial map shows the signals before and after enhancement and noise removal for spatial omics features. Abbreviation: principal component (PC).

Using the representation framework of SpaGFT (Fig. 2a), the mathematical formulation of SVG identification can be derived as a k-bandlimited signal recognition problem, which determines the first k low-frequency FMs to best approximate the original graph signal (Fig. 2b refer to S1 of Supplementary Note 1). This formulation can overcome three main limitations of SVG identification methods: (*i*) no pre-assumption of regular patterns in model design (e.g., radial hotspot, curve belt, or gradient streak)^9^; (*ii*) interpretable representation of SVG patterns^18^ with spatial context; and (*iii*) high computational efficiency^19^ when processing large-scale datasets. Essentially, we defined and implemented a GFTscore for each gene to quantify the contribution of low-frequency FMs by determining the first k low-frequency FMs, weighting, and summing corresponding FCs (S2 of Supplementary Note 1). Based on the definition, a gene is identified as an SVG if (*i*) its GFTscore is greater than the inflection point based on the distribution of all genes’ GFTscore and (*ii*) its FCs of the first k low-frequency FMs are significantly higher than FCs of high-frequency FMs (S3 of Supplementary Note 1). Consequently, we evaluated the performance of SVG identification using 31 public SRT datasets from human and mouse brains (Supplementary Table 1)^20–23^. As no golden-standard SVG database was available, we collected 849 SVG candidates from five existing studies^23–27^, and 458 of them were used as curated benchmarking SVGs based on cross-validation with the In Situ Hybridization (ISH) database of Allen Brain Atlas (Supplementary Tables 2 and 3, Methods). The SVG prediction performance of SpaGFT was compared with SPARK^9^, SPARK-X^19^, MERINGUE^28^, SpatialDE^11^, and SpaGCN^4^, in terms of six reference-based and two reference-free metrics (Supplementary Note 2). The grid search of parameter combinations was conducted on three high-quality brain datasets to evaluate each tool’s performance, in which SpaGFT showed the highest median and peak scores (Fig. 2c and Supplementary Table 4). In addition, the computational speed of SpaGFT was two-fold faster than that of SPARK-X and hundreds-fold faster than those of the other four tools on the two Visium datasets (Supplementary Table 5). Although SpaGFT was slower than SPARK-X on the Slide-seqV2 dataset, it showed a remarkably enhanced SVG prediction performance compared to SPARK-X. We then performed an independent test on 28 independent datasets using the parameter combination with the highest median Jaccard Index among three datasets from the above grid-search test. The results revealed that SpaGFT promised supreme performance among the investigated tools based on the evaluation metrics (Fig. 2d, Supplementary Figs. 2a–d, and Supplementary Table 6). Within the top 500 SVGs from each of the above six tools, SpaGFT identified SVGs shared with other tools and also unique SVGs that were validated as the ground truth (Supplementary Fig. 2e and Supplementary Table 7).

For example, *Nsmf* and *Tbr1* were identified by all six tools and showed clear structures of the hippocampus, cortical region, and cerebral cortex. On the other hand, *Cartpt*, *Cbln2*, *Ttr*, and *Pmch* were uniquely identified by SpaGFT and showed key functions in the brain, such as *Cartpt* participating in dopamine metabolism (Fig. 2e, Supplementary Figs. 3a–d, and Annotation 1 of Supplementary Note 3). These benchmarking results suggested that SpaGFT is capable of leveraging upon the FM representation of gene expression for robust and accurate identification of SVGs from SRT data. SpaGFT takes advantage of FM representation of gene expression patterns in SVG identification, and the SVGs identified by SpaGFT were distinguishably separated from non-SVGs on the FM-based UMAP with a clear boundary, whereas SVGs were irregularly distributed on the principal component-based gene UMAP (Fig. 2f).

In addition, the distorted graph signal correction can be used as the mathematical formulation to impute a low-expressed gene or denoise a high-intensity but noisy protein in SpaGFT. Essentially, FCs are shifted towards a specific bandwidth by implementing a low-pass filter and are inversely transformed to an enhanced graph signal using an inverse graph Fourier transform (iGFT)^29^. To enhance the main signal and mitigate noise, a low-passing filter is employed to weigh and shift all FCs toward the low-frequency bandwidth (Methods). In the end, these weighted FCs are transformed back to a corrected graph signal via iGFT (Supplementary Fig. 4a). In assessing the performance of gene expression correction, we used 16 human brains SRT datasets with well-annotated spatial domains^22, 23^ and utilized adjusted rand index (ARI) to measure the accuracy of predicting spatial domains using corrected gene expression. As a result, SpaGFT outperformed other gene enhancement tools in terms of ARI, including Sprod^8^, SAVER-X, scVI, netNMF-sc, MAGIC, and DCA^30, 31^ (Fig. 2g, Supplementary Fig. 4b, and Supplementary Table 8). For example, SpaGFT enhanced the low-intensity spatial omics signal broadly across different technologies and species, such as gene *TNFRSF13C* for human lymph node, gene *Ano2* for mouse brain (Supplementary Fig. 4c), cell density for human prostate tumor (from data of Fig. 1k), protein I-A, and corresponding gene *H2ab1* for mouse breast tumor. Similarly, *the* noisy background can also be removed, such as protein LY6A/E and corresponding gene *Ly6a* and protein CD19 (Figs. 2h–i and Annotation 2 of Supplementary Note 3).

### SpaGFT identifies the germinal center, T cell zone, B cell zone, and crosstalking regions in the human lymph node.

**Figure 3.**
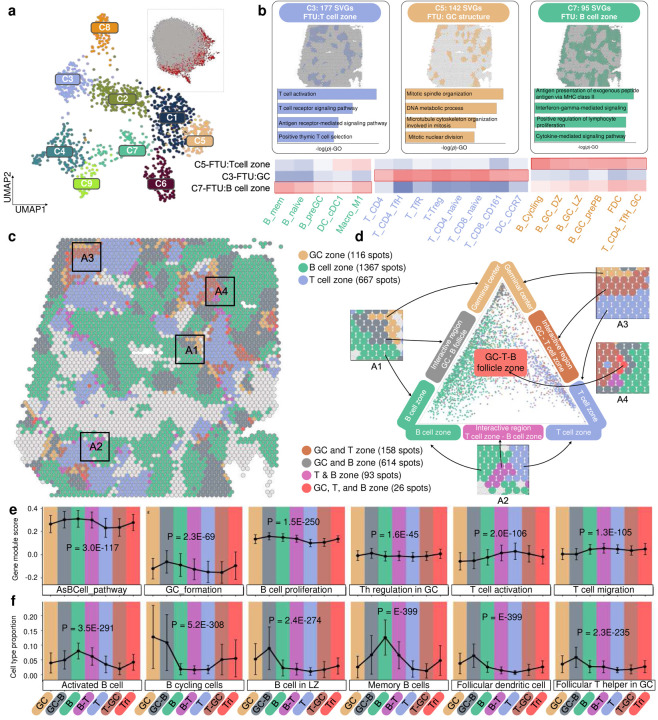
**a.** UMAP visualization of nine SVG clusters from the human lymph node. Each dot represented SVGs. Upright UMAP showed SVGs in red and non-SVG in gray. **b**. Clusters 3, 5, and 7 were highly associated with the T cell zone, GC, and B follicle cell components based on molecular and functional signatures. The heatmap visualized the FTU-cell type correlation matrix. **c**. The spatial map overlaid three FTUs and displayed the overlapped spots and unique spots. As different colors corresponded to spots, we selected four areas to showcase the region-to-region interaction. A1 showcased GC, GC-B interaction region, and B follicle. A2 showcased the B follicle, B-T interaction region, and T cell zone. A3 showcased the GC, GC-T interaction zone, and T cell zone. A4 displayed a B-GC-T interaction zone. **d**. The barycentric coordinate plot shows cell-type components and the abundance of spots in interactive and functional regions. If the spot is closer to the vertical of the equilateral triangle, the cell type composition of the spot tends to be signature cell types of the functional region. The spots were colored by functional region and interactive region categories. **e** and **f**. The three plots displayed changes in enriched functions and cell type components across seven regions (GC, GC-B, B, B-T, T, T-GC, T-GC-B). The P-value was calculated using one-way ANOVA to test the differences among the means of seven regions.

As low-frequency FC can represent smooth spatially variable patterns, they can be used for SVG clustering, and gene clusters can correlate with distinct FTUs from a gene perspective (Supplementary Fig. 5a). To demonstrate the application, we implemented SpaGFT in the publicly available Visium data of human lymph nodes, which, as secondary lymphoid organs contain well-known recurrent functional regions, such as T cell zones, B cell zones, and germinal center (GC)^18^. First, SpaGFT identified 1,346 SVGs and characterized nine SVG clusters (Fig. 3a and Supplementary Table 9). To recognize the FTUs of the T cell zone, B cell zone, and GC, we first used cell2location^32, 33^ to determine the cell proportion (Supplementary Fig. 5b and Supplementary Table 10) for the nine SVG clusters and investigate function enrichment (Supplementary Figs. 5c–e) for three selected FTUs. Based on the molecular, cellular, and functional signatures of three regions^32^, we found that SVG clusters 3, 5, and 7 (Fig. 3b) were associated with the T cell zone, GC, and B cell zone, respectively (Annotation 3 of Supplementary Note 3).

In contrast to spatial domain detection tools, SpaGFT is not restricted to a rigid boundary for tissue-level identification of microenvironments^5^. Instead, SpaGFT allows overlapping regions to infer the functional coherence and collaboration among different FTUs. We therefore projected three FTUs represented by SVG clusters 3, 5, and 7 on the spatial map for visual inspection, and identified their close spatial proximity (Fig. 3c). These results are highly indicative of tissue regions of polyfunctionality amongst these three TFUs (four representative subregions are shown in Fig. 3c). To further investigate the crosstalk amongst these three TFUs, we projected spots (assigned to all three regions) to the Barycentric coordinates (the equilateral triangle in Fig. 3d), which displayed relations and abundance of the unique and overlapped regions regarding cell type components^34^. We identified 614 spots overlapped with B cell zone and GC, 158 spots overlapped with GC and T zone, 93 spots overlapped with T zone and B cell zone, and 26 spots overlapped across three FTUs (Supplementary Table 11), in support of the complex interactions within these three TFUs. We next hypothesized that the spots from the overlapped region would vary in functions and cell components to support the polyfunctionality of these regions. We thus investigated the changes in enriched functions (Supplementary Table 12) and cell types (Supplementary Fig. 6) across seven regions (i.e., GC, GC-B, B, B-T, T, T-GC, and T-B-GC). Our results identified lymph node-relevant pathways and cell types, such as B and T cell activity and functions, as significantly varied across those regions (Figs. 3e–f, Annotation 4 of Supplementary Note 3), in support of our hypothesis.

### SpaGFT reveals secondary follicle variability based on CODEX data.

**Figure 4.**
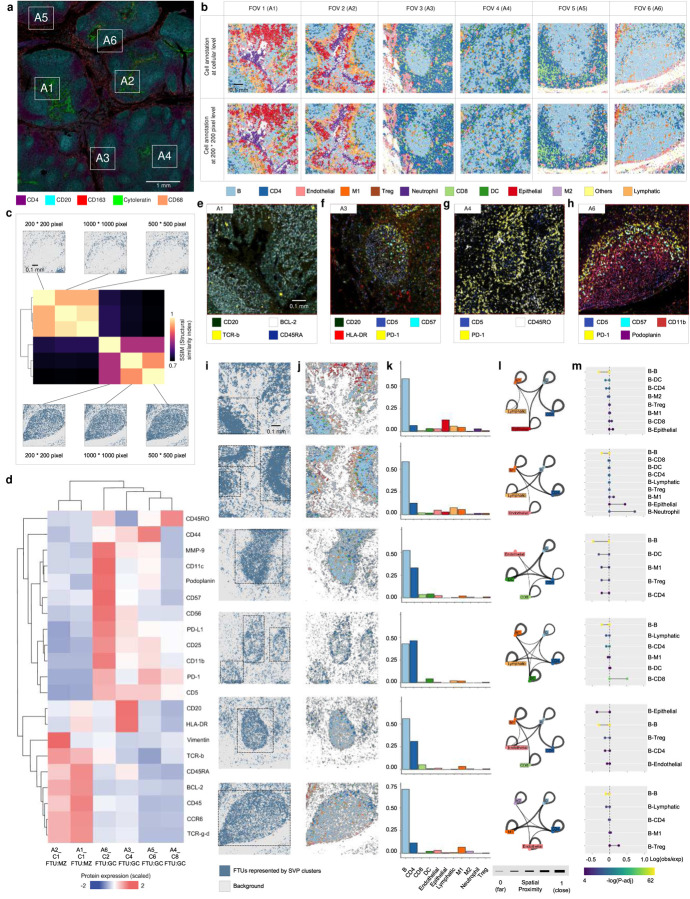
**a.** A 49-plex CODEX data was generated from human tonsil tissue at a 0.37μm/pixel resolution. Six FOVs were selected based on their varying tissue microenvironment and cellular organization. **b**. Cell phenotype maps for each of the six FOVs, depicting the cellular composition and organization. **c**. The results showed the characterization FTUs based on the gradient pixel-level images for A6. The heatmap depicts the SSIM score, where a higher score corresponds to a lighter color and greater structural similarity. **d**. A heatmap showcasing the protein expression of each FTUs represented by the six SVP clusters, which were identified as FTUs resembling secondary follicles. The values in the heatmap are scaled by z-scores of protein expression. **e-h**. Overlays of CODEX images for SVPs for FOVs 1, 3, 4, and 6, respectively. **i**. Spatial maps depicting the patterns of secondary follicle FTUs from six FOVs. Dash rectangles indicate the identified follicle regions. Note that panels d to h are ordered by FOV 1, 2, 3, 4, 5, and 6. **j**. Cell phenotype maps of the FTUs identified in **i**. **k**. Barplots depicting the cell components of the identified FTU in i. The cell type colors were depicted in **b**. **l**. The graph network depicting the spatial proximity of the top 5 abundant cell types in the FTU identified in i, as calculated by $\frac{1}{1+d}$, where d represents the average distance between any two cell types. m. Dumbbell plots indicated significant cell-cell interaction among B cells and others. If the observed distance is significantly smaller than the expected distance, the two cell types tend to be contacted and interact. Line length represents relative distances, subtracting the expected distance from the observed distance. The point size was scaled by adjusted p-value.

The results of Visium in Fig. 3 showcased the ability of SpaGFT to identify FTUs via SVG clustering. Given that the current resolution of Visium (~50μm per pixel) limited our ability to interpret the variability of finer follicle structures and their corresponding functions at the cellular level, we next performed single-cell level spatial proteomics on a human tonsil using a 49-plex CODEX panel at a ~0.37μm per pixel resolution (Fig. 4a) to better characterize and interpret the follicle variability we observed and inferred using SpaGFT on the Visium data. Based on the anatomical patterns highlighted by B (e.g., CD20) and T cell (e.g., CD4) lineage markers, we selected fields of view (FOV) that would allow for a good representation of the complex tissue structures present in the tonsil (i.e., GC and interfollicular space^35^) while still highlighting the variability in follicle structure^36^. We first performed cell segmentation with DeepCell^37^, followed by clustering with FlowSOM^38^ and Marker Enrichment modeling^39^ to identify the diverse cell phenotypes present in the data (Fig. 4b). Interestingly, we observed that the clear arrangement of T and B cell patterns (e.g., A3, A5, and A6) informed identifiable GC regions within the follicular structure, compared to others (e.g., A4) without clear T and B cell spatial organization (Fig. 4b). We, therefore, postulated that A4 is comprised of multiple follicles, unlike A5 and A6, to represent a more spatially complex FOV.

We investigated this further by directly using the raw CODEX images as inputs to identify FTUs formed from spatially variable protein (SVP) clusters within the tissue environment^40^. To verify whether downsampling the CODEX image (Supplementary Fig. 7a) would result in a loss in the power of characterizing FTUs, we first used FOV 6 to generate three images across different resolutions (with downsampling), resoluting in a 1) 1,000-by-1,000 pixel image (~0.8μm per pixel size), 2) 500-by-500 pixel image (~1.6μm per pixel size), and 3) 200-by-200 pixel image. Our results show that despite the generation of diverse low- and high-frequency FMs from three pixel-level images (as illustrated in Supplementary Fig. 7b), SpaGFT was stable to resolution changes, characterizing FTUs across different resolutions with consistent patterns (Supplementary Fig. 7c). We subsequently calculated the structural similarity score (SSIM) to quantitatively evaluate pattern similarity among identified FTUs. Each gradient pixel size image identified six FTUs, and those patterns of FTUs showed pairwise consistency (Fig. 4c and Supplementary Fig. 7d), suggesting that 200-by-200 pixel downsampled images (an approximate factor of 105 fold from the original pixel size) were sufficient in characterizing FTUs to balance between computational efficiency and biological insights.

We next implemented SpaGFT to characterize FTUs for the six FOVs with 200-by-200 pixel images and annotated follicles for each FOV based on cell components (Supplementary Fig. 8) and protein signatures (Supplementary Table 13; Supplementary Figs. 9a–b). Specifically, FTUs represented by SVP cluster 1 of A1 and SVP cluster 1 of A2 displayed the morphological features akin to that of a mantle zone (MZ). Molecularly, we uncovered that the B cell-specific marker^41^ (CD20) and anti-apoptotic factor (BCL-2)^42^ were SVPs for these two FTUs of A1 and A2 (Figs. 4d–e and Supplementary Fig. 9c). Our results confirmed the presence of CD20 in delineating the MZ structure, and additionally suggest that the presence of BCL-2 as a novel additional feature of MZ structures^43^. In another case, FTUs represented SVP cluster 4 of A3, SVP cluster 9 of A4, and SVP cluster 4 of A5 displayed GC-specific T cell signatures (Figs. 4f–4g and Supplementary Fig. 9d) and corresponding molecular features, including PD-1^44^ and CD57^45^, indicating the presence of well-characterized GC-specific T follicular helper cells^46^. For FTUs represented by SVP cluster 2 of A6, we observed a complex molecular environment, where Podoplanin, CD11c, and CD11b were SVPs, thus showcasing the existence of follicular dendritic cell (FDC)^47^ and GC-centric macrophages^48^ networks (Fig. 4h). In addition to molecular heterogeneity, we further captured their variability in terms of length-scale and morphology (Fig. 4i), cell type (Figs. 4j and k), cell-cell distance (Fig. 4l), and cell-cell interactions (Fig. 4m). For example, from the tissue morphology perspective, A3, A4, A5, and A6 captured clear oval shape patterns with different length-scales, but A1 and A2 captured multiple partial MZ patterns (Fig. 4i). Although visual inspection was unable to distinguish between the morphological patterns of GCs in A4 (Fig. 4b), SpaGFT was able to determine three small length-scale GC patterns at the molecular level (Fig. 4i).

Regarding cellular characteristics, six FTUs (i.e., two MZ from A1 and A2; four GCs from A3 to A6) were dominated by B and CD4 T cells with varying proportions (Figs. 4j–k; Supplementary Table 14). Specifically, MZs from A1 and A2 showed an average composition of 58% B and 10% CD4 T cells. GC from A3 and A5 with similar length-scale showed an average of 54% B and 32% CD4 T cells. A4 captured three length-scale GCs and showcased 43% B and 46% CD4 T cells, while the large-scale GC from A6 contained 70% B and 12% T cells, indicating B and T cell proportions varying in different length-scale GC. We could also infer cell-cell interaction based on distance (Figs. 4l–m). In general, MZ from A1 and A2 show that the observed B-B distance was smaller than the expected distance, which suggests the homogeneous biological process of the significant B-B interaction in the GC region. In addition, cell-cell interaction also shows heterogeneity for two MZ. The interactions between CD4 T cells and B cells were observed in two MZ from A1 and A2, showcasing the infiltration of CD4 T cells into the B cell right mantle zone^49^. DC-B and CD4 T-B cell interactions in A3 and A4 suggest light zone functions for B cell selection^50, 51^. Macrophage-B cell interactions in GC in A6 potentially indicated macrophage regulation on B cells (e.g., B cells that failed to trigger the cell proliferation signals during the B cell selection process, underwent apoptosis, and were subsequently engulfed by macrophages^52^). Our results demonstrate the applicability of SpaGFT at an initial subsampled lower resolution from high-plex spatial proteomics, thus efficiently identifying and characterizing high-attention tissue regions, including secondary follicles, to uncover cellular and molecular variability that can be further confirmed at the original single-cell resolution. We also affirmed that FTUs identified by SpaGFT were not simply regions of cell aggregation, but reflected both the cellular and regional activity and cell-cell interactions based on spatially orchestrated molecular signatures.

### SpaGFT can generate new features and be implemented as an explainable regularizer for machine learning algorithms.

SpaGFT can also be beneficial to enhance the performance of existing methods as an explainable regularizer through feature or objective engineering. To elucidate its applicative power, we exemplified three representative analyses of SRT as follows (Supplementary Fig. 10 and Methods).

**Figure 5.**
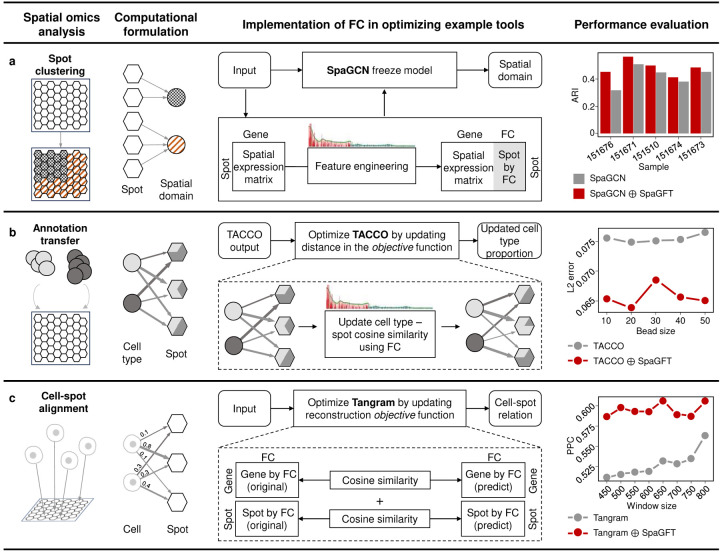
SpaGFT implementation for three cell-centric tools and the figure consists of four columns, each corresponding to spatial omics analysis, computational formulation, implementation of FC in optimizing examples tools, and performance evaluation. **a**. The first column shows spot clustering concepts. In this bipartite graph (second column), spot clustering can be formulated as a many-to-one mapping problem, where the source node represents the spot, the target node represents the spatial domain, and the edges denote the corresponding mapping relationships. Regarding the modified workflow of SpaGCN (third column), we changed the original input of SpaGCN. In addition to the spatial expression matrix with rows representing spots and columns representing cells, we computed the spot-spot similarity and applied SpaGFT to form a spot-by-FC matrix (Methods). This can be interpreted as project spot-spot similarity in the frequency domain. Following this, we concatenated the original expression matrix and the spot by FC matrix to generate a new input matrix. This newly formed matrix was then placed into the frozen SpaGCN model for computation. The top 5 performance-increased samples are distinctly showcased, where the y-axis is the ARI value, and the x-axis is the sample number (fourth column). Higher ARI indicates better performance. **b**. The first column shows annotation transfer concepts. In this bipartite graph (second column), annotation transfer is formulated as a many-to-many mapping problem, where the source node represents the annotation (e.g., cell types (CT)), the target node represents spots, and the edges represent the proportion from the source to the target node. Notably, the sum of the weights of all edges pointing to the same target node is equal to 1. Regarding the modified workflow of TACCO (third column), we made modifications to the cost matrix in optimal transport. Originally, the cost matrix was calculated using genes as features to measure the cosine similarity between CT and spots, thereby measuring the distance from cell to spot. In the new cost matrix calculation method, we use weighted FCs as the feature to calculate the distance between CT and spots and then optimize the baseline mapping matrix (e.g., TACCO output). In the evaluation (fourth column), we refer to TACCO methods to simulate spots with different bead sizes using scRNA-seq data and use L2 error to measure differences between predicted and known cell composition in each simulated spot. The y-axis is the bead size for a simulation data value, and the x-axis is the L2 error. Lower L2 error scores indicates better performance. **c**. The first column shows cell-spot alignment concepts. In this bipartite graph (second column), the cell-spot alignment can be formulated as a many-to-many mapping problem, where the source node represents a single cell, the target node represents spots, and the edges represent the probability of alignment from the source to the target node. Unlike annotation transfer, the sum of the weights of all edges emanating from the same source node equals 1. Regarding the modified workflow of Tangram (third column), we have added two additional constraint terms to the original objective function of Tangram. The first constraint is designed from a gene-centric perspective, calculating the cosine similarity of the gene by FC matrix between the reconstructed and the original matrix. The second constraint is designed from a cell-centric perspective, calculating the cosine similarity on the spot by the FC matrix between the reconstructed and the original matrix. In the evaluation (fourth column), we first refer to the previous studies’ method to simulate spatial gene expression data using different window sizes based on STARmap data. Subsequently, we measure the similarity between predicted and known cell proportions in each simulated spot using the Pearson correlation coefficient. Higher PPC indicates better performance.

First, we showcase how spot clustering can identify spatial domains spatially coherent in both gene expression and histology. Here, we selected SpaGCN^4^ as the demonstration to showcase the implementation of FC from the feature engineering aspect (Fig. 5a). To illustrate FCs being a novel feature, we extended the spatial expression matrix by concatenating a spot-by-FC matrix derived from the spot-spot similarity. Subsequently, the new feature matrix was input into the original SpaGCN model and predicted spatial domains. Same as the SpaGCN study, we utilized 12 datasets^23^ of human brain SRT data for training (two datasets from the same tissue section) the number of new features and testing for improving SpaGCN on ten datasets. The results indicated improvements in eight out of ten datasets (Supplementary Table 15) in identifying the spatial domains of the dorsolateral prefrontal cortex. Notably, the top five datasets exhibited enhancements between 7.8% and 42.6%.

Second, annotation transfer will solve the challenge of insufficient data labeling for the increasing emergence of SRT. We used TACCO^6^ as an annotation transfer example tool to showcase the application of FC as a regularizer for the optimal transport (OT) method, which is a machine learning method that aimed to find the most efficient way (i.e., minimizing the overall cost associated with the movement) to move a probability distribution from one configuration to another. Specifically, TACCO allowed the transfer of phenotype-level annotation labels (e.g., cell type) from scRNA-seq to SRT using such an OT framework. Although TACCO has demonstrated algorithm effectiveness in consideration of cell similarity over all genes, we hypothesized that projecting cell similarity to the frequency domain and strengthening a topological regularization in OT’s objective function will be a potential avenue for performance enhancement. In our modification, we integrated a topological regularization term into the original cost matrix to derive a new cost matrix (Fig. 5b and Methods). Leveraging the evaluation metrics of the original TACCO study, our tests underscored an 8.7% to 14.9% L2-error decrease across five simulated bead sizes in terms of transferring annotated labels from scRNA-seq to unannotated SRT mouse brain data (Supplementary Table 16).

Third, aligning single-cell data (e.g., scRNA-seq) to low-/high-resolution SRT data was important to mutually benefit each other regarding spatial resolution and molecular diversity. We selected Tangram^7^ as an alignment tool to demonstrate the topological regulation of genes and spots in the frequency domain. Tangram optimized the cell-to-spot mapping matrix through the gradient-based method, aiming to ensure the similarity between the reconstructed SRT based on ScRNA-seq and the original SRT. The objective function of Tangram is to measure cell density, gene-level similarity, and cell-level similarity in the vertex domain, respectively. In alignment with the hypothesis proposed in Fig. 5b, we constrained the similarity at both the gene- and cell-level in the frequency domain (Fig. 5c). As a result, our tests illustrated 7.4%~15.9% Pearson correlation coefficient increase improvement regarding aligning scRNA-seq on simulated STARmap^53^ mouse brain SRT data (Supplementary Table 17).

### SpaGFT introduces an inductive bias to regularize the deep learning method and identify rare subcellular organelles.

**Figure 6.**
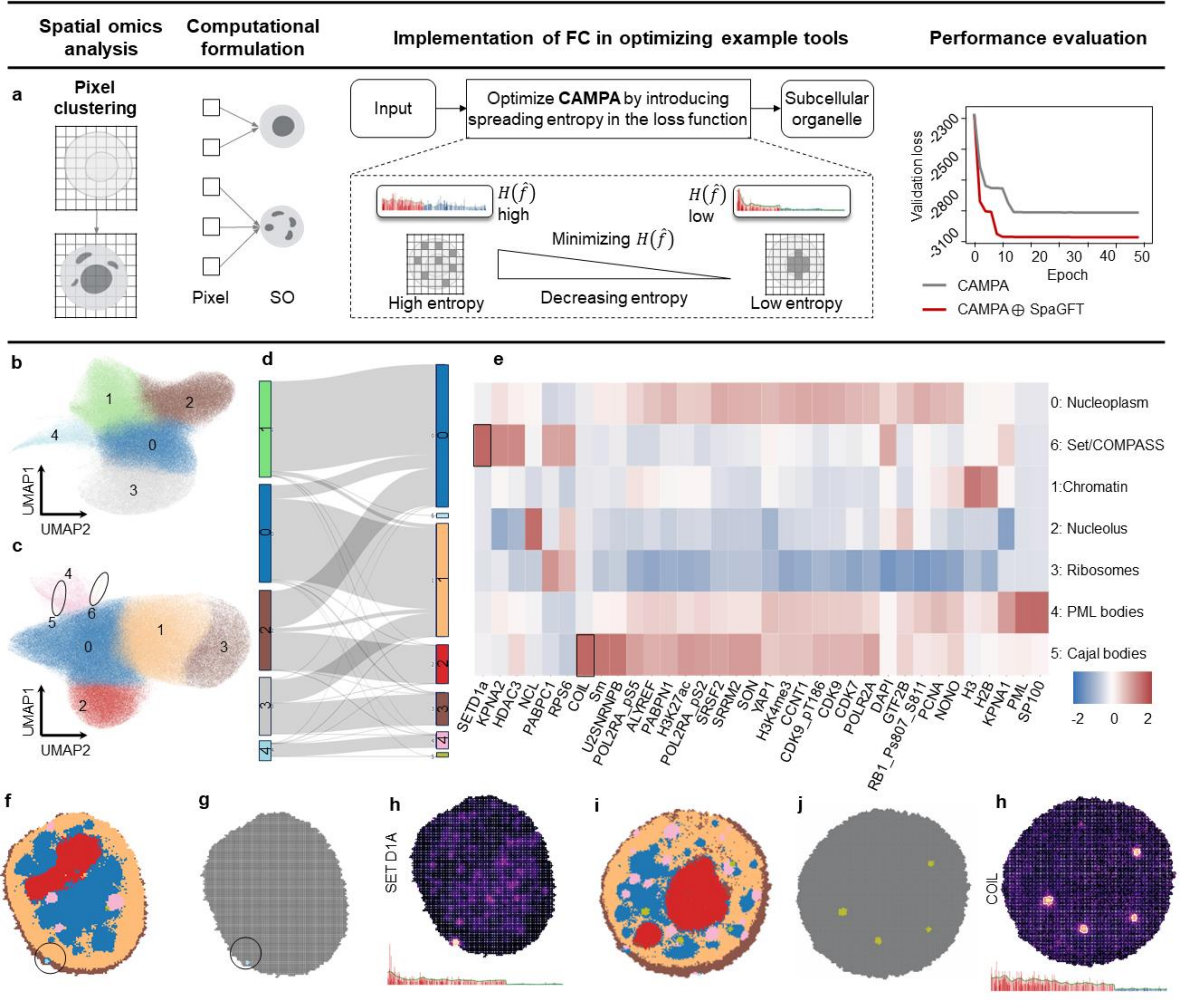
The case for implementing FC to CAMPA. **a**. The first column shows pixel clustering concepts. In this bipartite graph (second column), pixel clustering can be formulated as a many-to-one mapping problem, where the source node represents the pixel, the target node represents the subcellular organelle, and the edges denote the corresponding mapping relationships. Regarding the modified workflow of CAMPA (third column), we have made a modification to the original loss function. The modified term aims to measure the spreading of graph signals in the reconstructed image. In the frequency domain, this spreading can be quantified using spreading entropy (Method). A spreading graph signal corresponds to high entropy, while a non-spread graph signal corresponds to low entropy. Therefore, the new regularizer term aims to minimize the spreading entropy. In the evaluation (fourth column), we used the validation loss, which was calculated using the same loss function and validation dataset to examine the contribution of the spreading entropy to the model training. The y-axis is the validation loss value, and the x-axis is the number of epochs for training the CAMPA model. **b**. UMAP shows five pixel clusters predicted by the baseline model using the Leiden clustering algorithm at 0.2 resolution. **c**. UMAP shows seven pixel clusters predicted by the modified model using the Leiden clustering algorithm at 0.2 resolution. Two rare clusters were circled in this panel. **d**. The Sanky plot shows the cluster changes from baseline model prediction to modified model prediction. **e**. The heatmap shows the annotation of each cluster (modified model at resolution 0.2) using a human protein atlas. The column of the heatmap is the protein intensity in the cell nucleus, and the row corresponds to clusters. **f**–**h**. The three figures showcase the overview of predicted pixel clusters, cluster 6, and marker protein for cell 224081. **i**–**h**. The three figures showcase the overview of predicted pixel clusters, cluster 5, and marker protein for cell 367420.

We applied SpaGFT to obtain an interpretable spreading entropy regulation for a conditional variational autoencoder framework, CAMPA, to identify conserved subcellular organelles across multiple perturbed conditions on pixel-level 4i data (165 *nm*/pixel)^15, 54^. To modify the model, we introduced an entropy term in the original reconstruction loss of CAMPA to regularize the spreading of graph signals^17^. Specifically, we constrained the entropy within the first k bandwidth and provided an inductive assumption for CAMPA to learn embeddings that represented k-bandlimited signals (Supplementary Fig. 11a). Consequently, compared to the validation loss calculated from validation datasets (Methods), the loss curve from the modified model showed a reduction and entered a stable state earlier (Fig. 6a). We observed that, by introducing the entropy term as a regularizer, the model enhanced the training efficacy in capturing and minimizing the reconstruction error and promoted faster convergence of the model.

Furthermore, we validated that the modified model significantly (p-value = 0.035) improved the baseline model regarding batch effect removal (Supplementary Figs. 11b–e) using kBET testing^55^, indicating that the learned embeddings retained conserved structures of subcellular organelles across multiple perturbations. Next, compared with the baseline (Figs. 6b–d), the modified model additionally identified two rare clusters (Supplementary Table 18), including cluster 5 (with an average of 0.16% pixels per cell) and cluster 6 (with an average of 0.10% pixels per cell). Notably, the pixels assigned to these two clusters are very stable (not random signals computationally) regardless of different resolution parameters of the Leiden clustering algorithm (Supplementary Table 19 and Supplementary Fig. 11f). Subsequently, clusters 5 and 6 were annotated as Cajal bodies^56^ and set1/COMPASS^57^, respectively (Fig. 6e). Cluster 6 and its corresponding protein signature, SETD1A (Figs. 6f–h), displaying a highly concentrated pattern (with an average of 0.16% pixels per cell), were strongly shown as a k-bandlimited signal in the frequency domain. Furthermore, we also observed similar characteristics of cluster 5 and the corresponding marker protein, COIL (Figs. 6i–h). Therefore, we concluded that by introducing regularization of low-frequency signals in SpaGFT, the model stably learned robust embeddings inclined to represent subcellular organelles showing k-bandlimited characteristics.

## Discussion

SpaGFT provides a robust and unbiased feature representation through graph Fourier transform that enhances our biological understanding of complex tissues. This method aligns with the advanced analytical capabilities required to dissect the intricate spatial components of tissue biology, from subcellular to multicellular scales.

From a computational standpoint, SpaGFT redefines the identification of SVGs by conceptualizing them as k-bandlimited signals. This eliminates the need for pre-defined expression patterns and significantly improves computational efficiency, as demonstrated in the benchmarking across 31 human/mouse Visium and Slide-seq V2 datasets. Furthermore, implementing a low-pass filter and inverse GFT effectively impute low-expressed gene expression and denoise high-noisy protein intensity, leading to more precise spatial domain predictions, as showcased in the human dorsolateral prefrontal cortex. Notably, SpaGFT advances the interpretation of spatial omics data by enabling more accurate machine learning predictions. It has notably improved the performance of existing frameworks by 8%−40% in terms of the accuracy of spatial domain identification, lower error of annotation transfer from cell types to spots, the correctness of the cell-to-spot alignments, and the validation loss of subcellular hallmark inference, respectively. These enhancements are pivotal in characterizing complex biological structures, including identifying extremely rare subcellular organelles, thus providing deeper insights into the cellular machinery.

Regarding the biological implications, SpaGFT offers new perspectives on spatial biology questions. Specifically, by grouping SVGs identified by SpaGFT, we can uncover distinct FTU within organs. This has led to the identification of critical immunological regions in the human lymph node Visium data, enhancing our knowledge of B cell maturation and the polyfunctional areas it encompasses, such as the B cell zone, T cell zone, GC, B-T zone, GC-B zone, T-GC, and tri-zone Additionally, exclusive in-house CODEX data, SpaGFT has revealed secondary follicle differences in the morphology, molecular signatures, and cellular interactions in the human tonsil, offering a more nuanced understanding of B cell maturation. Additionally, SpaGFT introduces k-bandlimited signal entropy within the CAMPA framework. This has led to the groundbreaking identification of rare subcellular organelles, which are the Cajal body and the Set1/COMPASS complex. The former is integral to the regulation of gene expression, while the latter plays a critical role in epigenetic modifications. By enabling the investigation of these organelles with unprecedented detail, SpaGFT propels us closer to a comprehensive understanding of the spatial dynamics of gene expression and the epigenetic landscape within cells.

However, there is still room for improving prediction performance and understanding the FTU mechanism. First, SpaGFT discusses low and high-frequency signals in the frequency domain, but there is a lack of discussion on mid-frequency signals. This is due to the complexity of mid-frequency signals in representing spatial patterns and their biological interpretations, which will be addressed in our future work. Second, although the SpaGFT computation speed is very competitive, it can be further enhanced by reducing the computational complexity from $O\left(n^{2}\right)$ to O(n×log(n)) using fast Fourier transform algorithms^58^. Third, the alteration of the spot graph and FTU topology represents a potential challenge in identifying FTUs across spatial samples from different tissues or experiments, which results in diverse FM spaces and renders the FCs incomparable. This is similar to the “batch effect” issue in multiple single-cell RNA sequencing (scRNA-seq) integration analyses. One possible solution to this challenge is to embed and align spatial data points to a fixed topological space using machine learning frameworks, such as optimal transport. Another possibility is to use H&E images as a common reference for all to make the embedding tissue-aware. Fourth, SpaGFT implementation on the CODEX image relies on experts’ knowledge to pre-select functional regions. The future direction of analyzing multiplexed images is to develop a topological learning framework to automatically detect and segment functional objects based on SpaGFT feature representation. Overall, we believe the value of our study is to bring a new view for explainable artificial intelligence in spatial omics modeling, including multi-resolution spatial omics data integration and pattern analysis across spatiotemporal data^12^.

## Methods

We introduce Spatial Graph Fourier Transform (SpaGFT) to represent spatial omics features. The core concept of SpaGFT is to transform spatial omics features into Fourier coefficients (FC) for downstream analyses, such as SVG identification, expression signal enhancement, and topological regularization for other machine algorithms. SpaGFT framework provides graph signal transform and seven downstream tasks: SVG identification, gene expression imputation, protein signal denoising, spatial domain characterization, cell type annotation, cell-spot alignment, and subcellular landmark inference. The detailed theoretical foundation of k-bandlimited signal recognition can be found in Supplementary Note 1.

### Graph signal transform

#### K-nearest neighbor (KNN) Graph construction.

Given a gene expression matrix containing n spots, including their spatial coordinates and m genes, SpaGFT calculates the Euclidean distances between each pair of spots based on spatial coordinates first. In the following, an undirected graph G=(V,E) will be constructed, where $V=\left\{v_{1},v_{2},\ldots ,v_{n}\right\}$ is the node set corresponding to n spots; E is the edge set while there exists an edge $e_{ij}$ between $v_{i}$ and $v_{j}$ in E if and only if $v_{i}$ is the KNN of $v_{j}$ or $v_{j}$ is the KNN of $v_{i}$ based on Euclidean distance, where i,j=1,2,…,n; and i≠j. Based on the benchmarking, the default K is defined as $\sqrt{n}$. Note that all the notations of matrices and vectors are bolded, and all the vectors are treated as column vectors in the following description. An adjacent binary matrix $A=\left(a_{ij}\right)$ with rows and columns as n spots is defined as:

(1) $$a_{ij}=\left\{\begin{array}{ll} 1, & e_{ij}\in E\\ 0, & \text{ else.} \end{array}\right.$$

A diagonal matrix $D=\text{diag}\;\left(d_{1},d_{2},\ldots ,d_{n}\right)$, where $d_{i}=\sum_{j=1}^{n}\;a_{ij}$ represents the degree of $v_{i}$.

#### Fourier mode calculation.

Using matrices A and D, a Laplacian matrix L can be obtained by

(2) L=D-A.

L can be decomposed using spectral decomposition:

(3) $$L=U\Lambda U^{T}\;\Lambda =\text{diag}\left(\lambda_{1},\lambda_{2},\ldots ,\lambda_{n}\right),\;U=\left(\mu_{1},\mu_{2},\ldots ,\mu_{n}\right),$$

Where the diagonal elements of Λ are the eigenvalues of L with $\lambda_{1}\leq \lambda_{2}\leq \cdots \leq \lambda_{n}$, where $\lambda_{1}$ is always equal to 0 regardless of graph topology. Thus, $\lambda_{1}$ is excluded for the following analysis. The columns of U are the unit eigenvector of $L.\mu_{k}$ is the $k^{\text{th}}$ Fourier mode (FM), $\mu_{k}\in R^{n},k=1,\;2,\ldots ,n$, and the set $\left\{\mu_{1},\mu_{2},\ldots ,\mu_{k}\right\}$ is an orthogonal basis for the linear space. For $\mu_{k}=\left(\mu_{k}^{1},\mu_{k}^{2},\ldots ,\mu_{k}^{n}\right)$, where $\mu_{k}^{i}$ indicates the value of the $k^{\text{th }}$ FM on node $v_{i}$, the smoothness of $\mu_{k}$ reflects the total variation of the $k^{\text{th }}FM$ in all mutual adjacent spots, which can be formulated as

(4) $$\frac{1}{2}\sum_{v_{i}\in V}\;\sum_{v_{j}\in V}\;a_{ij}{\left(\mu_{k}^{i}-\mu_{k}^{j}\right)}^{2}.$$

The form can be derived by matrix multiplication as

(5) $$\frac{1}{2}\underset{v_{i}\in V}{\sum }\underset{v_{j}\in V}{\sum }a_{ij}{\left(\mu_{k}^{i}-\mu_{k}^{j}\right)}^{2}=\frac{1}{2}\left[\underset{v_{i}\in V}{\sum }d_{i}{\left(\mu_{k}^{i}\right)}^{2}-2\underset{v_{i}\in V}{\sum }\underset{v_{j}\in V}{\sum }a_{ij}\mu_{k}^{i}\mu_{k}^{j}+\underset{v_{j}\in V}{\sum }d_{j}{\left(\mu_{k}^{j}\right)}^{2}\right]\;=\underset{v_{i}\in V}{\sum }d_{i}{\left(\mu_{k}^{i}\right)}^{2}-\underset{v_{i}\in V}{\sum }\underset{v_{j}\in V}{\sum }a_{ij}\mu_{k}^{i}\mu_{k}^{j}\;=\mu_{k}^{T}D\mu_{k}-\mu_{k}^{T}A\mu_{k}\;=\mu_{k}^{T}L\mu_{k}\;=\lambda_{k}$$

where $\mu_{k}^{T}$ is the transpose of $\mu_{k}$. According to the definition of smoothness, if an eigenvector corresponds to a small eigenvalue, it indicates the variation of FM values on adjacent nodes is low. The increasing trend of eigenvalues corresponds to an increasing trend of oscillations of eigenvectors; hence, the eigenvalues and eigenvectors of L are used as frequencies and FMs in our SpaGFT, respectively. Intuitively, a small eigenvalue corresponds to a low-frequency FM, while a large eigenvalue corresponds to a high-frequency FM.

#### Graph Fourier transform.

The graph signal of a gene g is defined as $f_{g}=\left(f_{g}^{1},f_{g}^{2},\ldots ,f_{g}^{n}\right)\in R^{n}$, which is a n-dimensional vector and represents the gene expression values across n spots. The graph signal $f_{g}$ is transformed into a Fourier coefficient ${\overset{ˆ}{f}}_{g}$ by

(6) $${\overset{ˆ}{f}}_{g}=\left({\overset{ˆ}{f}}_{g}^{1},{\overset{ˆ}{f}}_{g}^{2},\ldots ,{\overset{ˆ}{f}}_{g}^{n}\right)=U^{T}f_{g}$$

In such a way, ${\overset{ˆ}{f}}_{g}^{k}$ is the projection of $f_{g}$ on FM $\mu_{k}$, representing the contribution of FM $\mu_{k}$ to graph signal $f_{g},k$ is the index of $f_{g}$ (e.g., k=1,2,…,n). This Fourier transform harmonizes gene expression and its spatial distribution to represent gene g in the frequency domain. The details of SVG identification using ${\hat{f}}_{g}$ can be found below.

### SVG identification

#### GFTscore definition.

We designed a *GFTscore* to quantitatively measure the randomness of gene expressions distributed in the spatial domain, defined as

(7) $$\text{GFTscore}\;\left(f_{g}\right)=\sum_{k=1}^{n}\;e^{-\lambda_{k}}{\tilde{f}}_{g}^{k},$$

where $\lambda_{k}$ is the pre-calculated eigenvalue of L, and the normalized Fourier coefficient ${\tilde{f}}_{g}^{k}$ is defined as:

(8) $${\tilde{f}}_{g}^{k}=\frac{\left|{\overset{ˆ}{f}}_{g}^{k}\right|}{\sum_{i=1}^{n}\;\;\left|{\overset{ˆ}{f}}_{g}^{i}\right|}.$$

The gene with a high *GFTscore* tends to be SVG and vice versa. Therefore, all m genes are decreasingly ranked based on their *GFTscore* from high to low and denote these *GFTscore* values as $y_{1}\geq y_{2}\geq \cdots \geq y_{m}$. In order to determine the cutoff $y_{z}$ to distinguish SVG and non-SVGs based on *GFTscore*, we applied the Kneedle algorithm^59^ to search for the inflection point of a *GFTscore* curve described in Supplementary Note 1.

#### Wilcoxon rank-sum test implementation for determining SVGs.

Although the above GFTscore is an indicator to rank and evaluate the potential SVGs, a rigorous statistical test is needed to calculate the p-value for SVGs and control type I error. First, SpaGFT determines low-frequency FM and high-frequency FMs and corresponding FCs by applying the Kneedle algorithm to the eigenvalues of L. The inflection points are used for determining the low-frequency FMs and high-frequency FMs when the direction parameters are ‘increasing’ and ‘decreasing’, respectively.

Second, the Wilcoxon rank-sum test is utilized to test the differences between low-frequency FCs and high-frequency FCs to obtain statistical significance. If a gene has a high GFTscore and significantly adjusted p-value, the gene can be regarded as an SVG. We use $f_{1},f_{2},\ldots ,f_{n}$ to represent the expression of a random signal on n spots. $f_{i}$ is followed by Gaussian distributions and can be regarded as independent and identically distributed (*i.i.d*.) random variables^11^. By implementing GFT on $\left(f_{1},f_{2},\ldots ,f_{n}\right)$, we obtain Fourier coefficients $FC_{1},FC_{2},\cdots ,FC_{p}$, where p is the number of low-frequency FCs and reflects the contributions from low-frequency FMs. We also obtain the $FC_{p+1},FC_{p+2},\cdots ,FC_{p+q}$, where q is the number of high-frequency FCs and reflects the contributions from noise. Hence, we form the null hypothesis that no difference exists between low-frequency FCs and high-frequency FCs (**Proof can be found in** S3 of Supplementary Note 1). Accordingly, a non-parametrical test (i.e., Wilcoxon rank-sum test) is used for testing the difference between median values of low-frequency FCs and high-frequency FCs. Especially, the null hypothesis is that the median of low-frequency FCs of an SVG is equal to or lower than the median of high-frequency FCs. The alternative hypothesis is that the median of low-frequency FCs of a SVG is higher than the median of high-frequency FCs. The p-value of each gene is calculated based on *Wilcoxon* one-sided rank-sum test and then adjusted using the false discovery rate (FDR) method. Eventually, a gene with *GFTscore* higher than $y_{z}$ and adjusted p-value less than 0.05 is considered an SVG.

### Benchmarking data setup

#### Dataset description.

Thirty-two spatial transcriptome datasets were collected from the public domain, including 30 10X Visium datasets (18 human brain data, 11 mouse brain data, and one human lymph node data) and two Slide-seqV2 datasets (mouse brain). More details can be found in Supplementary Table 1. Those samples were sequenced by two different SRT technologies: 10X Visium measures ~55μm diameter per spot, and Slide-seqV2 measures ~10μm diameter per spot. Three datasets were selected as the training sets for grid-search parameter optimization in SpaGFT, including two highest read-depth datasets in Visium (HE-coronal) and Slide-seqV2 (Puck-200115–08), one signature dataset in Maynard’s study^23^. The remaining 28 datasets (excluding lymph node data) were used as independent test datasets.

#### Data preprocessing.

For all the 32 datasets, we adopt the same preprocessing steps based on *squidpy* (version 1.2.1), including filtering genes that have expression values in less than ten spots, normalizing the raw count matrix by counts per million reads method and implementing log-transformation to the normalized count matrix. No specific preprocessing step was performed on the spatial location data.

#### Benchmarking SVG collection.

We collected SVG candidates from five publications^23–27^, with data from either human or mouse brain subregions. (*i*) A total of 130 layer signature genes were collected from Maynard’s study^23^. These genes are potential multiple-layer markers validated in the human dorsolateral prefrontal cortex region. (*ii*) A total of 397 cell-type-specific (CTS) genes in the adult mouse cortex were collected from Tasic’s study (2016 version)^27^. The authors performed scRNA-seq on the dissected target region, identified 49 cell types, and constructed a cellular taxonomy of the primary visual cortex in the adult mouse. (*iii*) A total of 182 CTS genes in mouse neocortex were collected from Tasic’s study^26^. Altogether, 133 cell types were identified from multiple cortical areas at single-cell resolution. (*iv*) A total of 260 signature genes across different major regions of the adult mouse brain were collected from Ortiz’s study^24^. The authors’ utilized spatial transcriptomics data to systematically profile subregions and delivered the subregional genes using consecutive coronal tissue sections. (v) A total of 86 signature genes in the cortical region shared by humans and mice were collected from Hodge’s study^25^. Collectively, a total of 849 genes were obtained, among which 153 genes were documented by multiple papers. More details, such as gene names, targeted regions, and sources, can be found in Supplementary Table 2.

Next, the above 849 genes were manually validated on the *in-situ* hybridization (ISH) database deployed on the Allen Brain Atlas (https://mouse.brain-map.org/). The ISH database provided ISH mouse brain data across 12 anatomical structures (i.e., Isocortex, Olfactory area, Hippocampal formation, Cortical subplate, Striatum, Pallidum, Thalamus, Hypothalamus, Midbrain, Pons, Medulla, and Cerebellum). We filtered the 849 genes as follows: (*i*) If a gene is showcased in multiple anatomical plane experiments (i.e., coronal plane and sagittal plane), it will be counted multiple times with different expressions in the corresponding experiments, such that 1,327 genes were archived (Supplementary Table 3). (*ii*) All 1,327 genes were first filtered by low gene expressions (cutoff is 1.0), and the *FindVariableFeatures* function (“*vst*” method) in the Seurat (v4.0.5) was used for identifying highly variable genes across twelve anatomical structures. Eventually, 458 genes were kept and considered as curated benchmarking SVGs. The evaluation criteria can be found in Supplementary Note 2.

### SpaGFT implementation and grid search of parameter optimization

A grid-search was set to test for six parameters, including *ratio_neighbors* (0.5, 1, 1.5, 2) for KNN selection and S(4,5,6,8) for the inflection point coefficient, resulting in 16 parameter combinations. We set $K=\sqrt{n}$ as the default parameter for constructing the KNN graphs in SpaGFT. SVGs were determined by genes with high *GFTscore* via the *KneeLocator* function (curve=‘convex’, direction=‘deceasing’, and S = 6) in the *kneed* package (version 0.7.0) and FDR (cutoff is less than 0.05). Detailed implementation and tutorial can be found on SpaGFT GitHub: https://github.com/OSU-BMBL/SpaGFT.

### Parameter setting of other tools

(*i*) SpatialDE (version 1.1.3) is a method for identifying and describing SVGs based on Gaussian process regression used in geostatistics. *SpatialDE* consists of four steps, establishing the SpatialDE model, predicting statistical significance, selecting the model, and expressing histology automatically. We selected two key parameters, *design_formula* (‘0’ and ‘1’) in the *NaiveDE.regress_out* function and *kernel_space* (“{‘SE’:[5.,25.,50.],’const’:0}”, “{‘SE’:[6.,16.,36.],’const’:0}”, “{‘SE’:[7.,47.,57.],’const’:0}”, “{‘SE’:[4.,34.,64.],’const’:0}”, “{‘PER’:[5.,25.,50.],’const’:0}”, “{‘PER’:[6.,16.,36.],’const’:0}”, “{‘PER’:[7.,47.,57.],’const’:0}”, “{‘PER’:[4.,34.,64.],’const’:0}”, and “{‘linear’:0,’const’:0}”) in the *SpatialDE.run* function for parameter tunning, resulting in 18 parameter combinations.

- SPARK (version 1.1.1) is a statistical method for spatial count data analysis through generalized linear spatial models. Relying on statistical hypothesis testing, SPARX identifies SVGs via predefined kernels. First, raw count and spatial coordinates of spots were used to create the SPARK object via filtering low-quality spots (controlled by *min_total_counts*) or genes (controlled by *percentage*). Then the object was followed by fitting the count-based spatial model to estimate the parameters via *spark.vc* function, which is affected by the number of iterations (*fit.maxiter*) and models (*fit.model*). Lastly, ran *spark.test* function to test multiple kernel matrices and obtain the results. We selected four key parameters, *percentage* (0.05, 0.1, 0.15), *min_total_counts* (10, 100, 500) in *CreateSPARKObject* function, *fit.maxiter* (300, 500, 700), and *fit.model* (“poisson”, “gaussian”) in *spark.vc* function for parameter tuning, resulting in 54 parameter combinations.
- SPARK-X (version 1.1.1) is a non-parametric method that tests whether the expression level of the gene displays any spatial expression pattern via a general class of covariance tests. We selected three key parameters, *percentage* (0.05, 0.1, 0.15), *min_total_counts* (10, 100, 500) in the *CreateSPARKObject* function, and *option* (“single”, “mixture”) in the *sparkx* function for parameter tuning, resulting in 18 parameter combinations.
- SpaGCN (version 1.2.0) is a graph convolutional network approach that integrates gene expression, spatial location, and histology in spatial transcriptomics data analysis. *SpaGCN* consisted of four steps, integrating data into a chart, setting the graph convolutional layer, detecting spatial domains by clustering, and identifying SVGs in spatial domains. We selected two parameters, the value of ratio (1/3, 1/2, 2/3, and 5/6) in the *find_neighbor_cluster* function and *res* (0.8, 0.9, 1.0, 1.1, and 1.2) in the *SpaGCN.train* function for parameter tuning, resulting in 20 parameter combinations.
- MERINGUE (version 1.0) is a computational framework based on spatial autocorrelation and cross-correlation analysis. It composes of three major steps to identify SVGs. Firstly, Voronoi tessellation was utilized to partition the graph to reflect the length scale of cellular density. Secondly, the adjacency matrix is defined using geodesic distance and the partitioned graph. Finally, gene-wise autocorrelation (e.g., Moran’s I) is conducted, and a permutation test is performed for significance calculation. We selected *min.read* (100, 500, 1000), *min.lib.size* (100, 500, 1000) in the *cleanCounts* function and *filterDist* (1.5, 2.5, 3.5, 7.5, 12.5, 15.5) in the *getSpatialNeighbors* function for parameter tuning, resulting in 54 parameter combinations.

### Visualization of frequency signal of SVGs in PCA and UMAP

Mouse brain (i.e., HE coronal sample) with 2,702 spots was used for demonstrating FCs on distinguishing SVG and non-SVG in the 2D UMAP space. SpaGFT determined 207 low-frequency FMs using Kneedle Algorithm and computed corresponding FCs. PCA was also used for producing low-dimension representation. The transposed and normalized expression matrix was decomposed by using the *sc.tl.pca* function from the *scanpy* package (version 1.9.1). The first 207 principal components (PC) were selected for UMAP dimension reduction and visualization. The function *sc.tl.umap* was applied to conduct UMAP dimension reduction for FCs and PCs.

### SVG signal enhancement

An SVG may suffer from low expression or dropout issues due to technical bias^8^. To solve this problem, SpaGFT implemented the low-pass filter to enhance the SVG expressions. For an SVG with an observed expression value $f_{g}\in R^{n}$, we define ${\overset{-}{f}}_{g}\in R^{n}$ as the expected gene expression value of this SVG, and $f_{g}={\overset{-}{f}}_{g}+\epsilon_{g}$, where $\epsilon_{g}\in R^{n}$ represents noises. SpaGFT estimates an approximated FCs $f_{g}^{⋆}$ to expected gene expression ${\overset{-}{f}}_{g}$ in the following way, resisting the noise $\epsilon_{g}$. The approximation has two requirements (i) the expected gene expression after enhancement should be similar to the originally measured gene expression, and (ii) keep low variation within estimated gene expression to prevent inducing new noises. Therefore, the following optimization problem is proposed to find an optimal solution $f_{g}^{⋆}$ for ${\overset{-}{f}}_{g}$

(9) $$f_{g}^{⋆}=\text{argmi}n_{f}\left[\|f-{f_{g}\|}^{2}+c\frac{1}{2}\underset{v_{i}\in V}{\sum }\underset{v_{j}\in V}{\sum }a_{ij}{\left(f^{i}-f^{j}\right)}^{2}\right]\;=\text{argmi}n_{f}\left[\|f-{f_{g}\|}^{2}+cf^{T}Lf\right]$$

where ∥⋅∥ is the L2-norm, $f=\left(f^{1},f^{2},\ldots ,f^{n}\right)\in R^{n}$ is the variable in solution space, and i,j=1,2,…,n.c is a coefficient to determine the importance of variation of the estimated signals, and c>0. According to convex optimization, the optimal solution $f_{g}^{⋆}$ can be formulated as:

(10) $$2\left(f_{g}^{⋆}-f_{g}\right)+2cLf_{g}^{⋆}=0\;\Rightarrow \left(I+cL\right)f_{g}^{⋆}=f_{g}\;\Rightarrow \left(UU^{T}+cU\Lambda U^{T}\right)f_{g}^{⋆}=f_{g}\;\Rightarrow U\left(I+c\Lambda \right)U^{T}f_{g}^{⋆}=f_{g}\;\Rightarrow f_{g}^{⋆}=U{\left(I+c\Lambda \right)}^{-1}U^{T}f_{g}=U{\left(I+c\Lambda \right)}^{-1}{\hat{f}}_{g}$$

where $\Lambda =\text{diag}\;\left(\lambda_{1},\lambda_{2},\ldots ,\lambda_{n}\right)$, and I is an identity matrix. $(I+c\Lambda {)}^{-1}$ is the low-pass filter and $(I+c\Lambda {)}^{-1}{\overset{ˆ}{f}}_{g}$ is the enhanced FCs. $f_{g}^{⋆}=U(I+c\Lambda {)}^{-1}{\overset{ˆ}{f}}_{g}$ represents the enhanced SVG expression using inverse graph Fourier transform. Specifically, in HE-coronal mouse brain data analysis, we selected $1,300(=25\sqrt{n},n=2702)$ low-frequency FCs for enhancing signal and recovering spatial pattern by using iGFT with c=0.0001.

### Data preprocessing on the human prostate cancer Visium data

#### Cell segmentation.

The Visium image of human prostate cancer (adenocarcinoma with invasive carcinoma) from the 10X official website was cropped into patches according to spot center coordinates and diameter length. Each patch is processed by Cellpose for nuclei segmentation using the default parameter. Cell density in each patch is determined using the number of segmented cells.

#### Microbial alignment.

Following the previous tutorial^60^, the corresponding bam files were processed via Kraken packages by (1) removing host sequences and retaining microbial reads, (2) assigning microbial reads to a taxonomic category (e.g., species and genus), (3) computing the relative abundance of different species in each spot.

### SVG signal enhancement benchmarking

Sixteen human brain datasets with the well-annotated label were used for enhancement benchmarking^22, 23^. Samples 151510, 151672, and 151673 were used for grid search. Other 13 datasets were used for independent tests. SpaGFT can transform graph signals to FCs, and apply correspondence preprocessing in the frequency domain to realize signal enhancement of genes. Briefly, it is composed of three major steps. Firstly, SpaGFT is implemented to obtain FCs. Secondly, a low-pass filter is applied to weigh and recalculate FCs. Lastly, SpaGFT implements iGFT to recover the enhanced FCs to graph signals. We select c(0.003,0.005,0.007) and ratio_fms(13,15,17) in the low_pass_enhancement function, resulting in 9 parameter combinations. c = 0.005 and ratio_fms = 15 were selected for the independent test. For the parameters used for other computational tools, the details can be found as follows.

- *SAVER-X* (version 1.0.2) is designed to improve data quality, which extracts gene-gene relationships by adopting a deep auto-encoder and a Bayesian model simultaneously. SAVER-X is composed of three major steps roughly. Firstly, train the target data with an autoencoder without a chosen pretraining model. Secondly, filter unpredictable genes using cross-validation. Lastly, estimate the final denoised values with empirical Bayesian shrinkage. Two parameters were considered to explore the performance as well as the robustness of SAVER-X, including *batch_size* (32, 64, 128) in the *saverx* function and *fold* (4, 6, 8) in the *autoFilterCV* function, resulting in 9 parameter combinations.
- *Sprod* (version 1.0) is a computational framework based on latent graph learning of matched location and imaging data by leveraging information from the physical locations of sequencing to impute accurate SRT gene expression. The framework of Sprod can be divided into two major steps roughly, which are building a graph and optimizing objective function for such a graph to obtain the de-noised gene expression matrix. To validate its robustness, two parameters were adjusted, including *sprod_R* (0.1, 0.5) and *sprod_latent_dim* (8, 10, 12), to generate nine parameter combinations.
- *DCA* (version 0.3.1) is a deep count autoencoder network with specialized loss functions targeted to denoise scRNA-seq datasets. It uses the autoencoder framework to estimate three parameters (μ,θ,π) of zero-inflated negative binomial distribution conditioned on the input data for each gene. In particular, the autoencoder gives three output layers, representing for each gene the three parameters that make up the gene-specific loss function to compare to the original input of this gene. Finally, the mean (μ) of the negative binomial distribution represents denoised data as the main output. We set neurons of all hidden layers except for the bottleneck to (48, 64, 80) and neurons of bottleneck to (24, 32, 40) for parameter tuning, resulting in 9 parameter combinations.
- *MAGIC* (version 3.0.0) is a method that shares information across similar cells via data diffusion to denoise the cell count matrix and fill in missing transcripts. It is composed of two major steps. Firstly, it builds its affinity matrix in four steps which include a data preprocessing step, converting distances to affinities using an adaptive Gaussian Kernel, converting the affinity matrix A into a Markov transition matrix M, and data diffusion through exponentiation of M. Once the affinity matrix is constructed, the imputation step of MAGIC involves sharing information between cells in the resulting neighborhoods through matrix multiplication. We applied the *knn* settings (3, 5, 7) and the level of *diffusion* (2, 3, 4) in the MAGIC initialization function for parameter tuning, resulting in 9 parameter combinations.
- *scVI* (version 0.17.3) is a hierarchical Bayesian model based on a deep neural network, which is used for probabilistic representation and analysis of single-cell gene expression. It consists of two major steps. Firstly, the gene expression is compressed into a low-dimensional hidden space by the encoder, and then the hidden space is mapped to the posterior estimation of the gene expression distribution parameters by the neural network of the decoder. It uses random optimization and deep neural network to gather information on similar cells and genes, approximates the distribution of observed expression values, and consider the batch effect and limited sensitivity for batch correction, visualization, clustering, and differential expression. We selected *n_hidden* (64, 128, 256) and *gene_likelihood* (‘zinb’, ‘nb’, ‘poisson’) in the model.SCVI function for parameter tuning, resulting in 9 parameter combinations.
- netNMF-sc (version 0.0.1) is a non-negative matrix decomposition method for network regularization, which is designed for the imputation and dimensionality reduction of SCRNA-seq analysis. It uses a priori gene network to obtain a more meaningful low-dimensional representation of genes, and network regularization uses a priori knowledge of gene-gene interaction to encourage gene pairs with known interactions to approach each other in low-dimensional representation. We selected d (8, 10, 12) and alpha (80, 100, 120) in the netNMFGD function for parameter tuning, resulting in 9 parameter combinations.

### SVG clustering and FTU identification

The pipeline is visualized in Supplementary Fig. 5a. As the pattern of one SVG cluster can demonstrate specific functions of one FTU, the FTU may not necessarily display a clear boundary to its neighbor FTUs. On the contrary, the existence of overlapped regions showing polyfunctional regions is allowed. Computationally, the process of FTU identification is to optimize the resolution parameter of the Louvain algorithm for obtaining a certain number of biology-informed FTUs, which minimizes the overlapped area. Denote $G^{\prime }$ as the set of SVGs identified by SpaGFT. For each resolution parameter $\text{res}>0,G^{\prime }$ can be partitioned to $\left\{G_{1}^{\prime },G_{2}^{\prime },\ldots ,G_{n_{\text{res}}}^{\prime }\right\}$ (i.e., $\cup_{k}G_{i}^{\prime }=G^{\prime }$ and $G_{k}^{\prime }\cap G_{l}^{\prime }=\emptyset ,\forall k\neq l$.) by applying the Louvain algorithm on FCs, and the resolution will be optimized by the loss function below. Denote $X=\left(x_{s,g}\right)\in R^{|S|\times \left|G^{\prime }\right|}$ as the gene expression matrix, where S is the set of all spots. In the following, for each SVG group $G_{k}^{\prime },\text{pseudo}\;\left(s_{s,G_{k}^{\prime }}\right)=\sum_{g\in G_{k}^{\prime }}\;log\;\left(x_{s,g}\right)$ represents the pseudo expression value^4^ for spot i. Apply k-means algorithms with k=2 on $\{\text{pseudo}\left(s_{1,G_{k}^{\prime }}\right),\;\text{pseudo}\left(s_{2,G_{k}^{\prime }}\right),\ldots ,\text{pseudo}\left(s_{|S|,G_{k}^{\prime }}\right.$ to pick out one spot cluster whose spots highly express genes in SVG group $G_{k}^{\prime }$ and such spot cluster is identified as a FTU, denoted as $S_{i}\in S$. Our objective function aims to find the best partition of $G^{\prime }$ such that the average overlap between any two $S_{i},S_{j}$ is minimized:

$$\underset{\;\text{res}\;>0}{\text{argmin}}\frac{2\times \sum_{k\neq l}\;\;\left|S_{k}\cap S_{l}\right|}{n_{\text{res}\;}\times \left(n_{\text{res}\;}-1\right)}$$

### SpaGFT implementation on the lymph node Visium data and interpretation

#### Lymph node SVG cluster identification and FTU interpretation.

SVGs were identified on the human lymph node data (Visium) with the default setting of SpaGFT. To demonstrate the relations between cell composition and annotated FTUs, cell2location^32^ was implemented to deconvolute spot and resolve fine-grained cell types in spatial transcriptomic data. Cell2location was first used to generate the spot-cell type proportion matrix as described above, resulting in a cell proportion of 34 cell types. Then, pseudo-expression values across all spots for one FTU were computed using the method from the FTU identification section. Then, an element of the FTU-cell type correlation matrix was calculated by computing the Pearson correlation coefficient between the proportion of a cell type and the pseudo-expression of an FTU across all the spots. Subsequently, the FTU-cell type correlation matrix was obtained by calculating all elements as described above, with rows representing FTUs and columns representing cell types. Lastly, the FTU-cell type matrix was generated and visualized on a heatmap, and three major FTUs in the lymph node were annotated, i.e., the T cell zone, GC, and B follicle.

#### Visualization of GC, T cell zone, and B follicles in the Barycentric coordinate system.

Spot-cell proportion matrix was used to select and merge signature cell types of GC, T cell zone, and B follicles for generating a merged spot-cell type proportion matrix (an N-by-3 matrix, N is equal to the number of spots). For GC, B_Cycling, B_GC_DZ, B_GC_LZ, B_GC_prePB, FDC, and T_CD4_TfH_GC were selected as signature cell types. For T cell zone, T_CD4, T_CD4_TfH, T_TfR, T_Treg, T_CD4_naive, and T_CD8_naive were selected as signature cell types. For B follicle, B_mem, B_naive, and B_preGC were regarded as signature cell types. The merged spot-cell type proportion matrix was calculated by summing up the proportion of signature cell types for GC, T cell zone, and B follicle, respectively. Finally, annotated spots (spot assignment in Supplementary Table 11) were selected from the merged spot-cell type proportion matrix for visualization. The subset spots from the merged matrix were projected on an equilateral triangle via Barycentric coordinate project methods^34^. The projected spots were colored by FTU assignment results. Unique and overlapped spots across seven regions (i.e., GC, GC-B, B, B-T, T, T-GC, and T-GC-B) from three FTUs were assigned and visualized on the spatial map. Gene module scores were calculated using the *AddModuleScore* function from the Seurat (v4.0.5) package. Calculated gene module score and cell type proportion were then grouped by seven regions and visualized on the line plot (Figs. 3e–f). One-way ANOVA using function *aov* in R environment was conducted to test the difference among the means of seven regions regarding gene module scores and cell type proportions, respectively.

### CODEX tonsil tissue staining

An FFPE human tonsil tissue (provided by Dr. Scott Rodig, Brigham and Women’s Hospital Department of Pathology) was sectioned onto a No. 1 glass coverslip (22×22mm) pre-treated with Vectabond (SP-1800–7, Vector Labs). The tissue was deparaffinized by heating at 70°C for 1 hr and soaking in xylene 2x for 15 min each. The tissue was then rehydrated by incubating in the following sequence for 3 min each with gentle rocking: 100% EtOH twice, 95% EtOH twice, 80% EtOH once, 70% EtOH once, ddH_2_O thrice. To prepare for Heat-Induced Antigen Retrieval (HIER), a PT module (A80400012, ThermoFisher) was filled with 1X PBS, with a coverslip jar containing 1X Dako pH 9 Antigen Retrieval Buffer (S2375, Agilent) within. The PT module was then pre-warmed to 75°C. After rehydration, the tissue was placed in the pre-warmed coverslip jar, then the PT module was heated to 97°C for 20 min and cooled to 65°C. The coverslip jar was then removed from the PT module and cooled for ~15–20 min at room temperature. The tissue was then washed in rehydration buffer (232105, Akoya Biosciences) twice for 2 min each then incubated in CODEX staining buffer (232106, Akoya Biosciences) for 20 min while gently rocking. A hydrophobic barrier was then drawn on the perimeter of the coverslip with an ImmEdge Hydrophobic Barrier pen (310018, Vector Labs). The tissue was then transferred to a humidity chamber. The humidity chamber was made by filling an empty pipette tip box with paper towels and ddH_2_O, stacking the tip box on a cool box (432021, Corning) containing a −20°C ice block, then replacing the tip box lid with a 6-well plate lid. The tissue was then blocked with 200 μL of blocking buffer.

The blocking buffer was made with 180 μL BBDG block, 10 μL oligo block, and 10 μL sheared salmon sperm DNA; the BBDG block was prepared with 5% donkey serum, 0.1% Triton X-100, and 0.05% sodium azide prepared with 1X TBS IHC Wash buffer with Tween 20 (935B-09, Cell Marque); the oligo block was prepared by mixing 57 different custom oligos (IDT) in ddH_2_O with a final concentration of 0.5 μM per oligo; the sheared salmon sperm DNA was added from its 10 mg/ml stock (AM9680, ThermoFisher). The tissue was blocked while photobleaching with a custom LED array for 2 hr. The LED array was set up by inclining two Happy Lights (6460231, Best Buy) against both sides of the cool box and positioning an LED Grow Light (B07C68N7PC, Amazon) above. The temperature was monitored to ensure that it remained under 35°C. The staining antibodies were then prepared during the 2 hr block.

DNA-conjugated antibodies at appropriate concentrations were added to 100 μL of CODEX staining buffer, loaded into a 50-kDa centrifugal filter (UFC505096, Millipore) pre-wetted with CODEX staining buffer, and centrifuged at 12,500 *g* for 8 min. Concentrated antibodies were then transferred to a 0.1 μm centrifugal filter (UFC30VV00, Millipore) pre-wetted with CODEX staining buffer, filled with extra CODEX staining buffer to a total volume of 181 μl, added with 4.75 μl of each Akoya blockers N (232108, Akoya), G (232109, Akoya), J (232110, Akoya), and S (232111, Akoya) to a total volume of 200 μl, then centrifuged for 2 min at 12,500 *g* to remove antibody aggregates. The antibody flow through (99μl) was used to stain the tissue overnight at 4°C in a humidity chamber covered with a foil-wrapped lid.

After the overnight antibody stain, the tissue was washed in CODEX staining buffer twice for 2 min each before fixing in 1.6% paraformaldehyde (PFA) for 10 min while gently rocking. The 1.6% PFA was prepared by diluting 16% PFA in CODEX storage buffer (232107, Akoya). After 1.6% PFA fixation, the tissue was rinsed in 1X PBS twice and washed in 1X PBS for 2 min while gently rocking. The tissue was then incubated in the cold (−20°C) 100% methanol on ice for 5 min without rocking for further fixation and then washed thrice in 1X PBS as before while gently rocking. The final fixation solution was then prepared by mixing 20 μL of CODEX final fixative (232112, Akoya) in 1000 μL of 1x PBS. The tissue was then fixed with 200 μL of the final fixative solution at room temperature for 20 min in a humidity chamber. The tissue was then rinsed in 1X PBS and stored in 1XBBS at 4°C prior to CODEX imaging.

A black flat bottom 96-well plate (07–200-762, Corning) was used to store the reporter oligonucleotides, with each well corresponding to an imaging cycle. Each well contained two fluorescent oligonucleotides (Cy3 and Cy5, 5 μL each) added to 240 μL of plate master mix containing DAPI nuclear stain (1:600) (7000003, Akoya) and CODEX assay reagent (0.5 mg/ml) (7000002, Akoya). For the first and last blank cycles, an additional plate buffer was used to substitute for each fluorescent oligonucleotide. The 96-well plate was securely sealed with aluminum film (14-222-342, ThermoFisher) and kept at 4°C prior to CODEX imaging.

### CODEX antibody panel

The following antibodies, clones, and suppliers were used in this study: BCL-2 (124, Novus Biologicals), CCR6 (polyclonal, Novus Biologicals), CD11b (EPR1344, Abcam), CD11c (EP1347Y, Abcam), CD15 (MMA, BD Biosciences), CD16 (D1N9L, Cell Signaling Technology), CD162 (HECA-452, Novus Biologicals), CD163 (EDHu-1, Novus Biologicals), CD2 (RPA-2.10, Biolegend), CD20 (rlGEL/773, Novus Biologicals), CD206 (polyclonal, R&D Systems), CD25 (4C9, Cell Marque), CD30 (BerH2, Cell Marque), CD31 (C31.3+C31.7+C31.10, Novus Biologicals), CD4 (EPR6855, Abcam), CD44 (IM-7, Biolegend), CD45 (B11+PD7/26, Novus Biologicals), CD45RA (HI100, Biolegend), CD45RO (UCH-L1, Biolegend), CD5 (UCHT2, Biolegend), CD56 (MRQ-42, Cell Marque), CD57 (HCD57, Biolegend), CD68 (KP-1, Biolegend), CD69 (polyclonal, R&D Systems), CD7 (MRQ-56, Cell Marque), CD8 (C8/144B, Novus Biologicals), collagen IV (polyclonal, Abcam), cytokeratin (C11, Biolegend), EGFR (D38B1, Cell Signaling Technology), FoxP3 (236A/E7, Abcam), granzyme B (EPR20129–217, Abcam), HLA-DR (EPR3692, Abcam), IDO-1 (D5J4E, Cell Signaling Technology), LAG-3 (D2G4O, Cell Signaling Technology), mast cell tryptase (AA1, Abcam), MMP-9 (L51/82, Biolegend), MUC-1 (955, Novus Biologicals), PD-1 (D4W2J, Cell Signaling Technology), PD-L1 (E1L3N, Cell Signaling Technology), podoplanin (D2–40, Biolegend), T-bet (D6N8B, Cell Signaling Technology), TCR β (G11, Santa Cruz Biotechnology), TCR-γ/δ (H-41, Santa Cruz Biotechnology), Tim-3 (polyclonal, Novus Biologicals), Vimentin (RV202, BD Biosciences), VISTA (D1L2G, Cell Signaling Technology), α-SMA (polyclonal, Abcam), and β-catenin (14, BD Biosciences).

### CODEX tonsil tissue imaging

The tonsil tissue coverslip and reporter plate were equilibrated to room temperature and placed on the CODEX microfluidics instrument. All buffer bottles were refilled (ddH_2_O, DMSO, 1X CODEX buffer (7000001, Akoya)), and the waste bottle was emptied before the run. To facilitate setting up of imaging areas and z planes, the tissue was stained with 750 μL of nuclear stain solution (1 μL of DAPI nuclear stain in 1500 μL of 1X CODEX buffer) for 3 min, then washed with the CODEX fluidics device. For each imaging cycle, three images that corresponded to the DAPI, Cy3, and Cy5 channels were captured. The first and last blank imaging cycles did not contain any Cy3 or Cy5 oligos, and thus are used for background correction.

The CODEX imaging was operated using a 20x/0.75 objective (CFI Plan Apo λ, Nikon) mounted to an inverted fluorescence microscope (BZ-X810, Keyence) which was connected to a CODEX microfluidics instrument and CODEX driver software (Akoya Biosciences). The acquired multiplexed images were stitched, and background corrected using the SINGER CODEX Processing Software (Akoya Biosciences). For this study, six independent 2,048×2,048 field-of-views (FOV) were cropped from the original 20,744×20,592 image. The FOVs were selected to include key cell types and tissue structures in tonsils, such as tonsillar crypts or lymphoid nodules.

### Cell segmentation

Custom ImageJ macros were used to normalize and cap nuclear and surface image signals at the 99.7th percentile to facilitate cell segmentation. Cell segmentation was performed using a local implementation of Mesmer from the DeepCell library (deepcell-tf 0.11.0)^37^, where the multiplex_segmentation.py script was modified to adjust the segmentation resolution (microns per pixel, mpp). model_mpp = 0.5 generated satisfactory segmentation results for this study. Single-cell features based on the cell segmentation mask were then scaled to cell size and extracted as FCS files.

### Cell clustering and annotation

Single-cell features were normalized to each FOV’s median DAPI signal to account for FOV signal variation, arcsinh transformed with cofactor = 150, capped between 1^st^ - 99^th^ percentile, and rescaled to 0–1. Sixteen markers (cytokeratin, podoplanin, CD31, aSMA, collagen IV, CD11b, CD11c, CD68, CD163, CD206, CD7, CD4, CD8, FoxP3, CD20, CD15) were used for unsupervised clustering using FlowSOM^38^ (66 output clusters). The cell type for each cluster was annotated based on its relative feature expression, as determined via Marker Enrichment Modeling^39^, and annotated clusters were visually compared to the original images to ensure accuracy and specificity. Cells belonging to indeterminable clusters were further clustered (20 output clusters) and annotated as above.

### SpaGFT implementation on tonsil CODEX data and interpretation

#### Resize CODEX images and SpaGFT implementation.

As each FOV consisted of 2,048 by 2,048 pixels (~0.4μm per pixel size), the CODEX image needed to be scaled down to 200 by 200 pixels (~3.2μm per pixel size) to reduce the high computational burden (Supplementary Fig. 7a). Therefore, original CODEX images (2,048 by 2,048 pixels) were resized to 200 by 200 images by implementing function “resize” and selecting cubic interpolation from the imager package (v.42) in R environments. SpaGFT was then applied to the resized images by following default parameters.

#### Structural similarity (SSIM) calculation.

The Structural Similarity (SSIM) score was a measurement for locally evaluating the similarity between two images regardless of image size^61^. The SSIM score ranged from 0 to 1; a higher score means more similarity between two images. It was defined as follows:

$$\text{SSIM}=l(x,y{)}^{a}\cdot c(x,y{)}^{\beta }\cdot s(x,y{)}^{\gamma }$$

x and y were windows with 8 by 8 pixels; $l(x,y)=\frac{2\mu_{x}\mu_{y}+C_{1}}{\mu_{x}^{2}+\mu_{x}^{2}+C_{1}}$ was the luminance comparison function for comparing the average brightness of the two images regarding pixels x and $y.C_{1}$ is constant, and α is the weight factor of luminance comparison. $c(x,y)=\frac{2\sigma_{x}\sigma_{y}+C_{1}}{\sigma_{x}^{2}+\sigma_{x}^{2}+C_{2}}$ was the contrast comparison function for measuring the standard deviation of two images. $C_{2}$ is constant, and β is the weight factor of contrast comparison. $s(x,y)=\frac{\sigma_{xy}+C_{3}}{\sigma_{x}\sigma_{y}+C_{3}}$ was the structure comparison by calculating the covariance between the two images. $C_{3}$ is constant, and γ is the weight factor of structure comparison.

#### Cell-cell distance and interaction analysis.

To compute cell-cell distance within one FTU, we first select cells assigned to each FTU. An undirected cell graph was then constructed, where the cell was a node and edge connected by every two cells defined by the Delaunay triangulation using *the deldir* function from the deldir package (v.1.0–6). Subsequently, the edge represented the observed distance between the connected two cells, and Euclidean distance was used for calculating the distance based on the previous study^62^. Lastly, the average distance among different cell types was computed by taking the average of the observed cell-cell distance to generate the network plot. Regarding the determination of the cell-cell interaction, the spatial location of cells assigned in each FTU was permutated and re-calculated cell-cell distance as expected distance. Based on the previous study, if the cell-cell distance was lower than 15 μm^63^ (~ 5 pixels in the 200 by 200-pixel image), the cells will contact and interact with each other. Wilcoxon rank-sum test was used for the computed p-value for expected distance and observed distance. If the expected distance was significantly smaller than the observed distance, it suggested that cells would interact with each other.

### SpaGFT implementation in SpaGCN

Let $X_{spa}$ be the SRT gene expression matrix with the dimension $n_{\text{spot}}\times n_{\text{gene}}$, in which $n_{\text{spot}}$ and $n_{\text{gene}}$ represent the numbers of spots and genes, respectively. Upon normalization, the spot cosine similarity matrix $X_{s}$ is computed by the formula $X_{s}=X_{spa}X_{spa}^{T}$, yielding a matrix with dimension $n_{\text{spot}}\times n_{\text{spot}}$. Denote $U=\left(\mu_{1},\mu_{2},\ldots ,\mu_{n_{FC}}\right)$, where each $\mu_{l}$ is the l-th eigenvector of the Laplacian matrix of the spatial graph and $n_{FC}$ is the number of Fourier coefficients. Hence, graph Fourier transform is implemented to transform $X_{S}$ into the frequency domain by:

$${\hat{X}}_{s}=U^{T}X_{s}$$

Subsequently, the newly augmented spot-by-feature matrix is obtained by concatenating SRT gene expression matrix $X_{spa}$ and transformed signal matrix ${\hat{X}}_{s}$:

$$X_{\text{new}\;}=\text{concat}\;\left(X_{\text{spa}},{\hat{X}}_{s}\right)$$

Finally, the matrix $X_{\text{new}}$ is inputted into SpaGCN as a replacement of the original gene expression matrix to predict the spatial domain cluster labels across all spots.

To evaluate the performance of such modification, 12 human dorsolateral prefrontal cortex of 10x Visium datasets were applied in benchmarking based on annotations from the initial study of SpaGCN^4^. The Adjusted Rand Index (ARI) was selected as the evaluation metric to measure the consistency between the predicted spot clusters and manually annotated spatial domain. The parameter *num_fcs*, which controlled the count of FCs, was determined by utilizing a grid search methodology executed on datasets 151508 and 151670. The search spanned a range of values from 600 to 1400, sampled per 100 steps. Upon analysis, the optimal parameter value was established at 1000 (Supplementary Table 15), while the other parameters were set to the default in SpaGCN. Next, the performance was compared on the 10 remaining datasets for the independent test.

### SpaGFT implementation in TACCO

SpaGFT was implemented to improve the performance of TACCO, which leveraged Optimal Transport (OT) to transfer annotation labels from scRNA-seq to spatial transcriptomics data. The core objective function of TACCO is denoted by a cost matrix $C=\left(c_{tb}\right)$ and a proportion matrix $=\left(\gamma_{tb}\right)$:

$$\Phi (\Gamma )=\sum_{tb}\;\gamma_{tb}c_{tb}$$

Specifically, $c_{tb}$ quantifies the cost that transports an object b to an annotation t. In TACCO, principal component analysis (PCA) was used to reduce the dimension of scRNA-seq and spatial transcriptomics gene expression matrices to the PC matrices by keeping the first 100 PCs, respectively. Subsequently, C is computed by calculating the Bhattacharyya coefficients between cell type-averaged scRNA-seq and spatial transcriptomics PC matrices. Finally, the OT’s optimization is solved by using the Sinkhorn–Knopp matrix scaling algorithm to yield a ‘good’ proportion matrix Γ.

For finding Γ, the cost matrix C plays the most important role in the OT’s optimization process. Based on the originally calculated C, an updated cost matrix $C^{\text{update}}$ considering spatial topology information is fused. To incorporate this topology information from the spatial data, the coordinates of spatial spots are used to construct a spatial graph, which is as the input with gene expression and initial TACCO-calculated mapping Γ, which represent cell-type proportions into SpaGFT for calculating FCs of genes and cell types (CT). Subsequently, these gene FCs matrices were weighted and averaged by spot expression value to obtain the spots’ FCs for obtaining spot level constraints. The cosine distance is calculated between the FCs of spatial spots and the FCs of cell types to create the updated CT-spot cost matrix $C^{\text{update}}$. The $C^{\prime }$ is a united cost matrix fused by C and $C^{\text{update}}$ with a balancing parameter β as:

$$C^{\prime }=\beta C+(1-\beta )C^{\text{update}}$$

This updated $C^{\prime }$ is then fed back into TACCO’s OT algorithm to predict revised cell type proportions for the spatial data. In addition, we used a simulated validation dataset with the setting of *bead size* = 5 to conduct a grid search on the input parameters S, the sensitivity in the Kneedle algorithm from SpaGFT, and β for determining these hyperparameters. While maintaining computational efficiency, we ascertained that the updated TACCO with β=0.8 and S=24 can achieve the best performance. Our experiments reveal that the updated TACCO, enriched with SpaGFT features, outperforms the baseline TACCO model in the simulated independent test dataset with the setting of *bead size* ∈[10,20,30,40,50].

### SpaGFT implementation in Tangram

Denote $X_{sc}$ as the gene expression matrix of ScRNA-seq with the dimension $n_{\text{cell}}\times n_{\text{gene}}$, in which $n_{\text{cell}}$ and $n_{\text{gene}}$ represent the numbers of cells and genes, respectively. $X_{spa}$ is the SRT gene expression matrix with dimension $n_{\text{spot}}\times n_{\text{gene}}$, and $n_{\text{spot}}$ represents the number of spots. Tangram aims to find a mapping matrix $M={\left(m_{ij}\right)}_{n_{\text{cell}}\times n_{\text{spot}}}$, where $0\leq m_{ij}\leq 1,\Sigma_{i}^{n_{\text{spot}}}\;m_{ij}=1$ and $m_{ij}$ reflects the probability of cell i mapping to spot j. Hence, $M^{T}X_{sc}$ can be treated as the reconstructed SRT gene expression matrix using scRNA-seq. Let $X_{re}=M^{T}X_{sc}$. The regularization part of the original objective function of Tangram is as follows:

$$\Phi (M)=w_{1}\sum_{k=1}^{n_{\text{gene}}}\;\text{cosine}\;\left(X_{re}^{\cdot ,k},X_{\text{spa}}^{\cdot ,k}\right)+w_{2}\sum_{j=1}^{n_{\text{spot}}}\;\text{cosine}\;\left(X_{re}^{j,\cdot },X_{\text{spa}}^{j,\cdot }\right)$$

where the first term describes the cosine similarity of gene k across all spots in reconstructed SRT gene expression matrix and real SRT gene expression matrix, weighted by $w_{1}$; and the second term describes the cosine similarity of spot j across all genes in reconstructed SRT gene expression matrix and real SRT gene expression matrix, weighted by $w_{2}$. By maximizing the objective function, the optimal mapping matrix $M^{*}$ can be obtained.

Denote $U=\left(\mu_{1},\mu_{2},\ldots ,\mu_{n_{FC}}\right)$, where each $\mu_{l}$ is the l-th eigenvector of the Laplacian matrix of the spatial graph and $n_{FC}$ is the number of Fourier coefficients. Hence, we can implement graph Fourier transform for genes by:

$${\hat{X}}_{\text{spa}}\;=U^{T}X_{\text{spa}}\;{\hat{X}}_{re}\;=U^{T}X_{re}$$

Therefore, both ${\hat{X}}_{spa}$ and ${\hat{X}}_{re}$ are the representations of genes in the frequency domain with the dimension $n_{FC}\times n_{\text{gene}}$. In addition, $X_{spa}^{\prime }=X_{spa}X_{spa}^{T}$ can be considered as the spot similarity matrix calculated by gene expression from real SRT data with dimension is $n_{\text{spot}}\times n_{\text{spot}}$. Similarly, $X_{re}^{\prime }=\left(M^{T}X_{sc}\right){\left(M^{T}X_{sc}\right)}^{T}$ represents the spot similarity matrix calculated by gene expression in reconstructed SRT data. In this way, we can implement graph Fourier transform for spots by:

$${\tilde{X}}_{\text{spa}}\;=U^{T}X_{\text{spa}}^{\prime }\;{\tilde{X}}_{re}\;=U^{T}X_{re}^{\prime }$$

Therefore, both ${\tilde{X}}_{spa}$ and ${\tilde{X}}_{re}$ are the new representations of spots in the frequency domain with the dimension $n_{FC}\times n_{\text{spot}}$. Therefore, we improved the objective function of Tangram by adding the similarity measurements of genes and spots in the frequency domain. The new objective function is:

$$\Phi (M)=w_{1}\sum_{k=1}^{n_{\text{gene}}}\;\;\text{cosine}\;\left(X_{re}^{\cdot ,k},X_{\text{spa}}^{\cdot ,k}\right)+w_{2}\sum_{j=1}^{n_{\text{spot}}}\;\;\text{cosine}\;\left(X_{re}^{j,\cdot },X_{\text{spa}}^{j,\cdot }\right)+w_{3}\sum_{k=1}^{n_{\text{gene}}}\;\;\text{cosine}\;\left({\hat{X}}_{re}^{\cdot ,k},{\hat{X}}_{\text{spa}}^{\cdot ,k}\right)\;+w_{4}\sum_{j=1}^{n_{\text{spot}}}\;\;\text{cosine}\;\left({\tilde{X}}_{re}^{j\cdot },{\tilde{X}}_{\text{spa}}^{j,\cdot }\right)$$

where $w_{1}$ weights similarities of genes in the vertex domain; $w_{2}$ weights similarities of spots in the vertex domain; $w_{3}$ weights the similarities of genes in the frequency domain and $w_{4}$ weights the similarities of spots in the frequency domain.

To evaluate the performance of such modification. We adopted the evaluation scheme from Bin Li et.al. study. In addition, we simulated this SRT dataset by ‘gridding’ a dataset (STARmap) using various window sizes (400,450,…,1200). In addition, simulated datasets of window sizes 400 and 1200 were used for grid search to determine the hyperparameters. In this way, $w_{3}$ and $w_{4}$ were set to 11 and 1, respectively, and other parameters (including $w_{1}$ and $w_{2}$) were the default parameters of Tangram. Our experiments reveal that the updated Tangram, enriched with SpaGFT features, outperforms the baseline Tangram model.

### SpaGFT implementation in CAMPA

Overall, the CAMPA framework, a conditional variational autoencoder for identifying conserved subcellular organelle on pixel-level iterative indirect immunofluorescence, was modified by adding an entropy term on its loss function to regularize graph signal (e.g., protein intensity) spreading or concentration. Specifically, compared with the baseline CAMPA loss function, which computed the mean squared error (MSE) loss for each pixel, the modified loss function additionally considered protein global spreading at the cell level.

#### Data preparation for model training, testing, and validation.

Following the baseline CAMPA paper and guidelines^54^, 292,548 (0.05% of full data) pixels datasets were down-sampled from processed cell nuclei of I09 (normal), I10 (Triptolide treatment), I11 (normal), and I16 (TSA treatment) wells based on 184A1 cell line. The training, testing, and validation data were set to 70%, 10%, and 20%, respectively.

#### Entropy regularization.

For cell i∈I, where I was the complete set of all cells in the down-sampled data, the corresponding original protein signatures in each cell were denoted as $X^{i}$ with the dimension $n_{\text{pixel}}\times n_{\text{channel}}$, where $n_{\text{pixel}}$ and $n_{\text{channel}}$ represented the number of pixels in one cell and the number of proteins, respectively. Similarly, ${\hat{X}}^{i}$ was denoted as reconstructed protein signatures for cell i. To measure the spreading of reconstructed protein signatures in the frequency domain, ${\hat{X}}^{i}$ and the coordinates of pixels were input into SpaGFT for computing the FC ${\hat{F}}^{i}$ with the dimension $n_{FC}\times n_{\text{channel}}$, in which $n_{FC}$ was the number of FC. Denote $U=\left(\mu_{1},\mu_{2},\ldots ,\mu_{n_{FC}}\right)$, where each $\mu_{k}$ was the k-th eigenvector of the Laplacian matrix of the spatial neighboring graph for cell i. Hence, FCs of reconstructed protein signatures for cell i was calculated by:

$${\hat{F}}^{i}=U^{T}{\hat{X}}^{i}$$

Subsequently, ${\hat{F}}^{i}=\left({\overset{ˆ}{f}}_{1}^{i},{\overset{ˆ}{f}}_{2}^{i},\ldots ,{\overset{ˆ}{f}}_{n_{FC}}^{i}\right)$ was used to calculate entropy by the entropy function, which regularized a concentrated graph signal^17,64^,

$$L_{\text{Entropy}}=-\sum_{i\in I}\;\sum_{k=1}^{n_{FC}}\;\frac{{\left|{\hat{f}}_{k}^{i}\right|}^{2}}{{∥{\hat{F}}^{i}∥}_{2}}\text{log}\;\frac{{\left|{\hat{f}}_{k}^{i}\right|}^{2}}{{∥{\hat{F}}^{i}∥}_{2}}$$

where $∥\cdot {∥}_{2}$ presents $L^{2}$-norm.

In addition, the η parameter was used as a weighting term to balance the initial loss function and the entropy-decreasing loss function, assigned with 0.3 as default. The formula of the modified loss function $L_{\text{modified}}$ was as follows:

$$L_{\text{modified}}=\eta \sum_{i}^{I}\;D\text{ln}\;\sigma +\frac{D}{2\sigma^{2}}\text{MSE}\left({\hat{X}}^{i},X^{i}\right)+(1-\eta )L_{\text{Entropy}}$$

Where D is a constant, which was used the same as the baseline mode (D=0.5). The initial decoder loss function was a part of the objective function in CAMPA, which used an analytical solution from σ-VAE^65^ to learn the variance of the decoder. The MSE and the logarithm of the variance were minimized through σ, which was a weighting parameter between the MSE reconstruction term and the KL-divergence term in the CAMPA objective function. There was an analytic solution to compute the value of σ:

$$\sigma^{*2}=\text{MSE}\;\left(X^{i},v^{i}\right)$$

$\sigma^{*2}$ was estimated value for $\sigma^{2}$ and $v^{i}$ presented the estimated latent mean for $X^{i}$.

Regarding the implementation, the training and testing datasets were selected to build the modified and baseline models, respectively. Subsequently, to fairly compare the two models’ training efficiency, the same validation dataset and initial loss were implemented to evaluate the convergence of validation loss.

To interpret the modified CAMPA training efficiency improvement regarding biological perspective, batch effect removal and prediction accuracy were evaluated. Regarding batch effect removal, a proportion of 1% of pixels were subsampled from prepared data. First, UMAP embeddings calculated from the CAMPA latent representations were generated to visualize the mixture of three perturbation conditions. To quantitatively compare the batch effect removal between the baseline and modified model, the kBET^55^ score was computed using the CAMPA latent representations across perturbation conditions. Following kBET suggestion, 0.5% pixels (~1500 pixels) were iteratively selected for calculating the kBET score (a higher rejection rate suggested a better batch effect removal result) 10000 times using 1 to 100 neighbors.

Subsequently, the CAMPA latent representations were clustered utilizing the Leiden algorithm^54^ at resolutions of 0.2,0.4,0.6,0.8,1.2,1.6, and 2.0. To understand the identity of each cluster predicted by the modified CAMPA under the resolution of 0.2, the protein intensities in each pixel cluster were visualized in the heatmap. Each pixel’s channel values were averaged at the cluster level and scaled by channel (column-level) z-score. Clusters were annotated based on the highest expressed markers and human protein atlas.

To evaluate the conserveness and homogeneity of the predicted cluster across different resolutions, we implemented high-label entropy to quantify the trend of diverging from one cluster into two clusters^66^. For example, at the resolution of 0.2, all pixels of cluster 6 predicted by the modified model were used to calculate entropy via a probability vector with two lengths. The first element of this vector was a percentage of pixels at the current resolution (i.e., 0.2), which tended to be the largest cluster at the next resolution (e.g., 0.4). The second element of this vector was the percentage of the rest of the pixels at the current resolution, which tended to be other clusters at the next resolution. The high-label entropy was repeatedly calculated on the same pixels of one cluster within/across baseline and modified model across gradient resolutions (i.e., 0.2, 0.4, 0.6, 0.8, 1.2, 1.6, and 2.0). To visualize intact cells and summarize the relation between pixel and cell in Supplementary Table 19, seven clusters predicted by the modified model based on resolution 0.2 were transferred to all pixels from full-size data via function *project_cluster_data* in the CAMPA package. The illustrated examples (id: 367420 and 224081) were extracted to calculate the FC of COIL and SETD1A and visualize.

## Supplementary Material

1

## Data Availability

All datasets from 10x Visium can be accessed from https://www.10xgenomics.com/products/spatial-gene-expression. Slide-DNA-seq data is downloaded from https://singlecell.broadinstitute.org/single_cell/study/SCP1278. Slide-TCR-seq data is downloaded from https://singlecell.broadinstitute.org/single_cell/study/SCP1348/slide-tcr-seq-data. The GSM5519054_Visium_MouseBrain data can be accessed via the GEO database with an accession number of GSM5519054. Regarding the human brain dataset, twelve samples can be accessed via endpoint “jhpce#HumanPilot10x” on Globus data transfer platform at http://research.libd.org/globus/. The other six human brain datasets (2–3-AD_Visium_HumanBrain, 2–8-AD_Visium_HumanBrain, T4857-AD_Visium_HumanBrain, 2–5_Visium_HumanBrain, 18–64_Visium_HumanBrain, and 1–1_Visium_HumanBrain) can be accessed via the GEO database with an accession number of GSE220442 and https://bmbls.bmi.osumc.edu/scread/stofad-2. The two Slide-seqV2 datasets are available as accession number SCP815 in the Single Cell Portal via the link https://singlecell.broadinstitute.org/single_cell. MERFISH data (Slice1_Replicate1-Vizgen_MouseBrainReceptor) can be downloaded from https://console.cloud.google.com/marketplace/product/gcp-public-data-vizgen/vizgen-mouse-brain-map?pli=1&project=vizgen-gcp-share. Xenium data (Rep1-Cancer_Xenium_HumanBreast) is downloaded from https://www.10xgenomics.com/products/xenium-in-situ/human-breast-dataset-explorer. Spatial-CITE-seq data can be accessed via the GEO database with an accession number of GSE213264. Spatial epigenome–transcriptome co-profiling data (spatial_ATAC_RNA_MouseE13) can be accessed via the GEO database with an accession number of GSE205055. The 184A1 datasets used to train modified CAMPA reported in this manuscript can be found at https://doi.org/10.5281/zenodo.7299516. SPOT data can be accessed via the GEO database with an accession number of GSE198353. CODEX tonsil data accessed via Zenodo https://zenodo.org/records/10433896.
