# Supplementary material for: Graph Fourier transform for spatial omics representation and analyses of complex organs
Source: Res Sq. 2024 Feb 15:rs.3.rs-3952048. Preprint. [Version 1] doi: 10.21203/rs.3.rs-3952048/v1 (PMC10896409; doi:10.21203/rs.3.rs-3952048/v1)
Supplement: 1 [file NIHPPrs3952048v1-supplement-1.pdf]

Supplementary information

Supplementary Note 1 | Theoretical foundation and rationale of SpaGFT design. This supplementary note describes (1) the biological rationale and mathematical formulation of SpaGFT; (2) SVG identification regarding group Fourier transforms, and (3) GTFscore design and statistical testing.

Supplementary Note 2 | Supplementary method for Kneedle algorithm and spatially variable gene benchmarking evaluation criteria. This supplementary note describes the detailed method of the Kneedle algorithm and the equation of evaluation criteria.

Supplementary Note 3 | Gene function annotation. This supplementary note includes six annotations for gene and protein interpretation.

Terminology Box 1 | Definition of terminologies used in SpaGFT. This terminology box describes frequently used terminology in this paper in terms of biological terminologies and computational terminologies.

Supplementary Tables

Supplementary Table 1 | Data information. The table includes information on 32 spatial transcriptome datasets from the public domain. The first column shows the data ID in the original paper or data source; the second column shows the use of the data (i.e., for grid-search optimization, independent test, or case study); the third column shows the sequencing platform; the fourth to the sixth columns show the sample information, including species, conditions, and tissue sources; the rest of the columns shows the statistical information of each data, including the number of spots, the number of genes, the number of total reads, the mean read per spot, the standard deviation of the number of reads per spot, the mean number of genes per spot, and the standard deviation of genes per spots.

Supplementary Table 2 | 849 SVG candidates collected from the public domain. The table collects 849 unique cell-type- or layer-specific markers from five different kinds of literature. The first column records the mouse gene symbol. The second column records the paper source. The third column records the experiment object in each gene, where "M," "H," and "M&H" represent mouse, human, and both. The fourth column records the human gene symbol. The fifth column records the original source in the paper for each gene, either figures or supplementary files.

Supplementary Table 3 | 458 curated benchmarking SVGs validated by the Allen Brain Atlas. The first six columns correspond to general information on gene identifiers, including gene symbol (mouse), gene symbol (human), UniqueID, probe name, plane, and the experiment ID in the ISH database. The ISH intensity on 12 brain regions was recorded from column G to Column R, respectively, including Isocortex, Olfactory area (OLF), Hippocampal formation (HPF), Cortical subplate (CTXsp), Striatum (STR), Pallidum (PAL), Thalamus (TH), Hypothalamus (HY), Midbrain (MB), Pons (P), Medulla (MY), and Cerebellum (CB). All the records were downloaded from the ISH database. Column S records the mean ISH intensity of 12 mouse brain regions. Column T records whether the gene is considered a curated benchmarking SVG in this paper.

Supplementary Table 4 | Grid-search of parameter combination for SVG prediction. The table records the details of the performance comparison in terms of the grid-search of parameter optimization. The first four columns correspond to sample ID, tested software, sequence technology, and parameter combinations. The rest of the columns record eight evaluation matrices, including the Jaccard index, Tversky index, the odds ratio of Fisher's exact test,

precision, recall, F1 score, Moran's I, and Geary's C. If an element in this table is "NA," the software shows an error or ran out of time (running time was greater than 48 hours) during testing.

Supplementary Table 5 | Running time of SpaGFT and other tools on the three grid-search test data. The table records the running time and memory cost of SpaGFT, SPARK, SPARK-X, MERINGUE, SpatialDE, and SpaGCN on the HE-coronal, 151673, and Puck-200115-08 datasets. All tools and experiments were carried out in the same computing environment introduced in Methods. Columns A and B show tool names and sample names; Column C and D records the running time with the unit as second (S) and $\log_{10}(S)$, respectively; Column E is memory cost with the unit as a megabyte. For any experiments that spent over 24 hours, we labeled them as "NA."

Supplementary Table 6 | SVG prediction performance on 28 independent test datasets using default parameters. The table records the details of the performance comparison in terms of the independent test. The first column indicates the dataset ID, corresponding to the Dataset ID in Supplementary Table 1. The second column shows eight evaluation matrices, including the Jaccard index, the Tversky index, the odds ratio of Fisher's exact test, precision, recall, F1 score, Moran's I, and Geary's C. The other columns are the software. If an element in this table is "NA," the software shows an error or runs out of time (running time was greater than 48 hours) during testing.

Supplementary Table 7 | Summary of top 500 genes identified by SpaGFT and the fix benchmarking tools. The table records the unique and consistent SVGs of the top 458 SVGs identified by six tools for mouse brain data (HE-coronal). The first column is the gene name; Columns B, C, D, E, F, and G are software names; The values in Columns B to G indicate whether the gene is identified by this tool. If the value is equal to 1, it means the gene is the output of the top 458 SVGs in this software, and vice versa; Column H is the sum of values from Columns B to G, indicating the consistency of identified genes (the higher value, the higher consistency). When the value in Column H is "1," it means that this gene is uniquely identified by one of the tools from Columns B to G; Column I indicates whether this SVG is from 458 ground truth.

Supplementary Table 8 | Gene enhancement results. The table demonstrates the performance of different tools, including Sprod, SAVER-X, scVI, netNMF-sc, MAGIC, and DCA, using 18 human datasets (2-3, 2-5, 2-8, 18-64, 1-1, T4857, 151507, 151508, 151509, 151510, 151669, 151670, 151671, 151672, 151673, 151674, 151675, and 151676). Samples 151510， 151672， and 151673 are used for grid search. Other 13 datasets are used for independent tests. The first column is the method name. The second column is the data usage. The third column is the sample ID. The fourth column is the parameter information for either grid search or independent test. The fifth column is the ARI score calculated by inputting the ground truth label and predicted label.

Supplementary Table 9 | SVG clustering results in the human lymph node data. The table records all SVGs predicted from SpaGFT on the human lymph node data. Column A is the gene name; Columns B-D are gene interpretations; Column E is the number of spots having this SVG expressed; Column F is the corresponding *GFTscore*; Column G is the ranking of GFTscore; Columns H and I are the *p*-value and *q*-value of SVG, respectively; Column J is the SVG cluster labels. SVGs are arranged based on the GFTscore from high to low.

Supplementary Table 10 | Deconvolution results for human lymph node sample. The table shows the proportions of 34 cell types calculated by cell2location. The first column is the spot ID of the mouse sample. The rest of the columns are the cell proportions in 34 cell types.

Supplementary Table 11 | TFU assignment to each spot from lymph node data in terms of GC, T cell zone, and B follicle. The table demonstrates GC, T cell zone, B follicle, and their interactive region assignment label. The first column is the spot ID. The second and third columns are spatial coordinates. The last column is the assignment label, where “0” is no assignment; “T.zone” is the spot assigned as T cell zone; “B.follicle” is the spot assigned as B follicle; “GC” is the spot assigned as germinal center; “T.zone-B.follicle” is the spot assigned as the interactive region between T cell zone and B follicle; “GC-T.zone” is the spot assigned as the interactive region between GC and T cell zone; “GC-B.follicle” is the spot assigned as the interactive region between GC and B follicle; “GC-T.zone-B.follicle” is the spot assigned as interactive region among GC, T zone, and B follicle.

Supplementary Table 12 | Pathway and corresponding gene signatures. The table documents the gene used for pathway enrichment from the GSEA database. The first column is gene names, and the second column is pathway names.

Supplementary Table 13 | SVP clustering result for six FOVs from CODEX. The table shows the SpaGFT prediction results. Column A is the protein name; Column B is the number of pixels, suggesting the number of pixels expressing this protein; Column C is GFTscore and shows low-frequency FM contributions; Column D is the GFT score rank; Colum E and F show the p-value and adjusted the p-value based on the false discovery rate method; Column G is the SVP cluster labels; Column H is the FOV indicator.

Supplementary Table 14 | Annotation and cell type proportions for FTUs of each FOV. The table shows the cell type proportions for each FTU. Column A is the cell type name, including B cells, CD4 cells, CD8 cells, Dendritic cells, endothelial cells, epithelial cells, lymphatic cells, macrophage 1, macrophage 2, Neutrophil, and Tregs; Column B is the number of pixels, suggesting the number of pixels presenting this cell type; Column C is the percentage of this cell type; Column D and Column E are the FOV and SVP cluster labels, respectively; Column F is the manual assignment and annotation of FTUs.

Supplementary Table 15 | Grid search and independent test of modified SpaGCN. The top table shows grid search results. The two samples, 151508 and 151670, from the same tissue section were selected to calculate ARI in terms of the gradient number of FCs. The row showed the number of FCs, and the column showed the ARI score. The bottom table shows independent tests for the other ten datasets using ARI measurement.

Supplementary Table 16 | Grid search and independent test of modified TACCO. The top table shows grid search results. One simulated dataset (base size = 5) was used to search parameter lambda (weighting regularizer term) and S (cutoff of *k*-bandlimited signal) regarding L2 errors. The bottom table shows independent tests for the other five datasets using L2 error measurement.

Supplementary Table 17 | Grid search and independent test of modified Tangram. The top table shows grid search results. Two simulated datasets (window size = 400 and 1,200) were used to search w3 and w4 (weighting for two regularizer terms) regarding the Pearson correlation coefficient (PCC). The bottom table showed independent tests for the other eight datasets using PCC measurement across 450, 500, 550, 600, 650, 700, 750, and 800 window sizes.

Supplementary Table 18 | The number of pixels/cells in cluster 5 and cluster 6. The first section of this table indicates the number of cells containing cluster 5 and cluster 6 in four wells across three perturbations. The second section of this table indicates the number of pixels in

cluster 5 and cluster 6. The third section of this table indicates the mean number of pixels of each cell in cluster 5 and cluster 6. Note: the parenthesis shows the percentage of cells/pixels in total cells/pixels.

Supplementary Table 19 | Divergence trend of pixels in cluster 5 and cluster 6 for different resolutions using the Leiden clustering algorithm. The top table (modified model) interprets the cluster 5 divergence trend regarding the number of pixels, ARI, and entropy from resolution 0.2 to the other six resolutions. Regarding the baseline model in the top table, the same measuring criteria are implemented to calculate the divergence trend of cluster 5 pixels from resolution 0.2 of the modified model to the other seven resolutions in the baseline model. The bottom table shows the same trends for cluster 6.

Supplementary Figures

Supplementary Fig. 1 | Technology summary. The panel illustrates common spatial omics technologies with detailed produce names, including both single-modality and multi-modality approaches.

Supplementary Fig. 2 | Performance Comparison. a-d. The two panels show Moran's I and Geary's C scores on the grid-search testing for the HE-coronal sample and independent test for 28 datasets. The boxplot indicates Moran's I and Geary's C score distribution for six tools' grid-search results, respectively. The Black line in the box indicates the median value. **e.** Shared SVGs among six computational tools using the HE-coronal dataset. The bottom upset plot indicates uniquely identified SVGs and overlapped SVGs, and the bar plot in the middle shows the corresponding SVG number. The ground truth ratio panel (top) demonstrates the proportion of ground-truth SVG among shared SVGs across six tools.

Supplementary Fig. 3 | ISH evidence of four SVGs. a-d. The ISH database webpage shows four major pieces of information, including experiment information (top left), ISH high-resolution image (right), 3D expression (middle left), and ISH intensity of 12 mouse brain regions (bottom). In addition, we used a dashed line to circle out ISH high-intensity regions on ISH high-resolution images.

Supplementary Fig. 4 | Workflow of SVG enhancement. a. The low SVG expression signal can be enhanced by a low-pass filter and iGFT using low-frequency FCs. **b.** Boxplot showcases the performance of SVG signal enhancement for g independent test using 151507, 151508, 151609, 151670, 151671, 151674, 151675, 151676, 18-64, 2-5, 2-8, T4875. The y-axis is the ARI value, and the y-axis is the imputation tool name. **c.** The ISH database webpage shows *Ano2* information for signal enhancement valuation.

Supplementary Fig. 5 | Framework of SVG clustering and FTU characterization and three FTUs interpretation. a. The figure shows the workflow of SVG clustering and FTU characterization: (i) enhanced FCs are utilized for clustering and generating SVG clusters based on the Louvain clustering algorithm using an initial resolution parameter (e.g., 0.1). (ii) SVGs in each SVG cluster are recovered to enhanced graph signals (i.e., enhanced SVGs' expression), and the pseudo-expression value of one SVG cluster is calculated by averaging these corresponding enhanced SVGs' expressions. (iii) the pseudo-expression of SVG clusters is used for characterizing FTU candidates by utilizing the k-means algorithm. (iv) All FTU candidates are used for pairwise calculating average overlapped spots; subsequently, the number of overlapped spots is optimized until SpaGFT finds a resolution to produce the minimal overlapped spots and eventually produce FTUs. **b**. The heatmap visualizes the transposed FTU-cell type correlation matrix (i.e., spots of one FTU in the column, the cell type in the row, and each element means

correlation of pseudo-expression value and cell type proportion across spots assigned in this FTU). According to the transposed FTU-cell type correlation matrix, SVG clusters 3, 5, and 7 correspond to the T cell zone, GC, and B follicle, respectively. **c-e**. The three figures showed the annotation of the T cell zone, GC, and B follicle, respectively. Each annotation figure displays fundamental information about each FTU, including an FTU spatial map, the number of SVGs, samples of SVG, functional enrichment tests (e.g., Biological Process 2021 and REACTOME 2022), and cell type compositions.

Supplementary Fig. 6 | Cell proportion changes across different regions. The figure shows the other lymph node-relevant cell type changes across seven different regions.

Supplementary Fig. 7 | Workflow of resizing CODEX image and gradient pixel image comparison. a. The original codex image has 2,048 by 2,048 pixels and is resized down to the 200-by-200 pixel image. The resized pixel image is used for FTU characterization based on the SpaGFT model. **b.** Low-frequency FMs and high-frequency FMs are visualized in terms of 1,000 by 1,000 pixel image, 500-by-500 pixel image, and 200-by-200 pixel image. **c.** The figure visualizes the comparison of the gradient pixel image. The zoom-in section shows the details of three gradient images. **d.** The heatmap shows the structural similarity (SSIM) score regarding all FTUs characterized based on three gradient images. The higher SSIM corresponds to the lighter color and indicates the high similarity of the two compared images.

Supplementary Fig. 8 | Overview of characterized TFUs. The figure shows the SVP clustering results for six FOVs. The first row is the pixel-level cell type annotation, corresponding to 200-by-200 pixels. The other rows showcase FTU and their cell type annotations. We label B-follicle, GC, and T zone for corresponding FTUs.

Supplementary Fig. 9 | Heterogeneous molecules of SVP clusters associated with mantle zone and GC. a. The binary heatmap shows FTU-associated spatially variable proteins (SVP). The pink color means the corresponding protein belongs to this FTU. **b.** The heatmap showed the scaled protein expression. Each block visualizes FTU morphology along with protein expression, and the red rectangle points out the FTU-associated SVP. **c** and **d**. Overlaid CODEX images visualize FTU using FTU-associated SVPs for FOV 2 and 5, respectively.

Supplementary Fig. 10 | The detailed workflow of FC implementation in different tools. a. The original input of SpaGCN was changed. The spatial expression matrix was defined as rows representing spots and columns representing cells. We first transposed and computed the spot-spot similarity for the spatial expression matrix. Then, SpaGFT was applied to generate a spot-by-FC matrix. Following this, we concatenated the original expression matrix and the spot by FC matrix to generate a new input matrix. This newly formed matrix was then placed into the frozen SpaGCN model for computation. **b**. To modify the workflow of TACCO, we changed the way to calculate the cost matrix in optimal transport. Originally, the cost matrix was calculated using genes as features to measure the cosine similarity between cell type (CT) and spots, thereby measuring the distance from cell to spot. By inputting TACCO's original output, which was a compositional matrix $\boldsymbol{\Gamma}$ (i.e., row represented CT; column represented spot; and element was transporting probability from CT to spot), $\boldsymbol{\Gamma}$ was computed using SpaGFT and obtained CT by FC matrix. The spatial expression matrix was also computed using SpaGFT and obtained gene by FC matrix. Subsequently, the gene by FC matrix was multiplied by the spatial expression matrix to produce spot by FC matrix. Lastly, the spot by FC matrix and CT by FC matrix were used to compute cosine similarity and obtain an updated cost matrix. $\boldsymbol{C}^{update}$. The updated cost matrix was used for optimizing OT and updating the original $\boldsymbol{\Gamma}$. **c**. To modify the workflow of Tangram, we have added two additional constraint terms to the original objective function of Tangram. The

first constraint is designed from a gene-centric perspective, calculating the cosine similarity of the gene by FC matrix between the reconstructed and the original matrix. The second constraint is designed from a cell-centric perspective, calculating the cosine similarity on the spot by the FC matrix between the reconstructed and the original matrix. Lastly, the two added regularizers were integrated into the original tangram objective function (T-obj). The updated objective function optimized the gradient descent process and computed cell-spot mapping probabilities.

Supplementary Fig. 11 | Case study of subcellular organelle interpretations. a. To modify the workflow of CAMPA, we added the entropy term as a regularizer into the original loss function. The modified term aims to measure the spreading of graph signals in the reconstructed image. A spreading graph signal corresponds to high entropy, while a non-spread graph signal corresponds to low entropy. Therefore, the new regularizer term aims to minimize the spreading entropy. Lastly, we implemented the spreading entropy regularizer and calculated embedding in the latent space. **b.** The UMAP shows the batch effect removal result using the baseline model. **c.** The UMAP shows the batch effect removal result using the modified model. **d**. The figure shows the boxplot of kBET results. The y-axis indicates the acceptance rate, which reflects whether to reject the null hypothesis. A higher acceptance rate refers to a better batch effect removal result. A two-sided Wilcox rank-sum test is used for calculating the p-value. **e**. the smoothed spline figure shows the acceptance rate changes across different neighbors of the KNN graph in the kBET algorithm. The higher position of the curve indicates the better batch effect removal in a local neighborhood view. **f**. The figure shows the agreement of Cluster 5 and Cluster 6 in different Leiden resolutions. For example, pixels in Cluster 5/Cluster 6 predicted from the modified model at resolution 0.2 are used to calculate entropy with other clustering results (e.g., modified model and baseline model) across the other resolutions.

Supplementary Files

This is a list of supplementary files associated with this preprint. Click to download.

- SupplementaryNote1.docx
- SupplementaryNote2.docx
- SupplementaryNote3.docx
- SupplementaryTable1.xlsx
- SupplementaryTable2.xlsx
- SupplementaryTable3.xlsx
- SupplementaryTable4.xlsx
- SupplementaryTable5.xlsx
- SupplementaryTable6.xlsx
- SupplementaryTable7.xlsx
- SupplementaryTable8.xlsx
- SupplementaryTable9.xlsx
- SupplementaryTable10.xlsx
- SupplementaryTable11.xlsx
- SupplementaryTable12.xlsx
- SupplementaryTable13.xlsx
- SupplementaryTable14.xlsx
- SupplementaryTable15.xlsx
- Supplementarytable16.xlsx
- Supplementarytable17.xlsx
- SupplementaryTable18.xlsx
- SupplementaryTable19.xlsx
- Supplementaryfigures.pdf
- TerminologyBox1.docx
- Supplementaryinformation.docx
